# Peptide-targeted cubosome and hexosome nanoassemblies mitigate mitochondrial dysfunction in a MitoPark model

**DOI:** 10.1038/s41392-026-02897-w

**Published:** 2026-08-04

**Authors:** Thelma Akanchise, Fucen Luo, Borislav Angelov, Yuru Deng, Takehiko Fujino, Angelina Angelova

**Affiliations:** 1 Université Paris-Saclay, CNRS, Institut Galien Paris-Saclay, Orsay, France; 2 Extreme Light Infrastructure ERIC, Dolni Brezany, Czech Republic; 3 Wenzhou Institute, University of Chinese Academy of Sciences, Wenzhou, China; 4 Wenzhou Yuanpai Biotechnology Co., Ltd, Wenzhou, China; 5 Institute of Rheological Functions of Food, Fukuoka, Japan

**Keywords:** Neurological disorders, Gene expression analysis, Biophysics

## Abstract

Mitochondrial dysfunction is a primary pathogenic mechanism underlying dopaminergic neuron loss in the nigrostriatal pathway in Parkinson’s disease (PD). To investigate mitochondrion-targeted therapeutic strategies, we utilized the MitoPark mouse model, in which mitochondrial transcription factor A (*Tfam*) is selectively ablated in midbrain dopamine neurons, resulting in progressive neurodegeneration. We designed multifunctional lyotropic liquid crystalline nanoparticles (LCNPs) of the cubosome and hexosome types for noninvasive nose-to-brain delivery. These nanocarriers were engineered with lipids essential for membrane integrity (plasmalogens and ω-3 polyunsaturated fatty acids (PUFAs)) and a nonlamellar structural lipid (monoolein). They coencapsulated the neuroprotective antioxidants ginkgolide B and quercetin. To facilitate neuronal targeting and uptake, the surface of the LCNP was modified by conjugation with pituitary adenylate cyclase-activating polypeptide (PACAP) and a rabies virus glycoprotein (RVG)-derived peptide-oleic acid (RVG-OL) conjugate. In vitro studies using differentiated SH-SY5Y cells subjected to oxidative stress demonstrated that the targeted LNPs enhanced cellular uptake and activated key neuroprotective signaling cascades, including AKT, ERK, and STAT3 phosphorylation. In vivo, intranasal administration of the optimized LNPs in MitoPark mice was associated with a trend toward the preservation of dopaminergic neuronal markers (such as tyrosine hydroxylase) and the regulation of mitochondrial-related proteins such as ATP5A1. Transcriptomic profiling revealed extensive molecular reprogramming. The peptide-functionalized LNPs upregulated genes enriched in mitochondrial biogenesis (*Ppargc1a* and *Pink1*) and survival (*Bcl2*) but downregulated the expression of neuroinflammatory mediators (*Il6*, *Nos2, Myd88, and Trem2*) and apoptotic effectors. These findings establish peptide-targeted, therapeutic lipid (plasmalogen/PUFA)-based nanoassemblies as a potent nonviral platform for noninvasive nose-to-brain delivery that may modulate mitochondrial- and neurodegeneration-related signaling pathways in a genetic model of PD.

## Introduction

Mitochondrial dysfunction is increasingly recognized as a primary pathogenic driver in Parkinson’s disease (PD), a neurodegenerative disorder characterized by the progressive loss of dopaminergic (DA) neurons in the substantia nigra pars compacta (SNc). Mitochondrial transcription factor A (*Tfam*) is central to mitochondrial genomic stability as a nuclear-encoded protein essential for the transcription, replication, and packaging of mitochondrial DNA (mtDNA) into nucleoids.^1–3^ Loss of *Tfam* function induces mtDNA instability, respiratory chain deficits, and bioenergetic failure, events that precede and drive the degeneration of DA neurons.^4^ Consequently, therapeutic strategies that can preserve or restore mitochondrial function offer a promising avenue for disease modification. However, the development of such therapies requires preclinical models that closely recapitulate these specific defects rather than acute toxin-induced neuronal damage.

To investigate mitochondrion-targeted therapeutic strategies, we utilized the MitoPark (MP) mouse model.^5,6^ The MitoPark animal model represents the gold standard for investigating mitochondrial pathology in PD. It is generated by the conditional ablation of the *Tfam* gene specifically in midbrain DA neurons using the Cre-loxP system.^5–7^ This “reverse genetic” mouse model mimics the fundamental features of human PD, including adult-onset neurodegeneration, progressive motor deficits, and the formation of intraneuronal inclusions.^8^ It has been emphasized that the MitoPark mouse provides a unique and rigorous platform for evaluating interventions designed to rescue bioenergetic failure and respiratory chain deficiency,^9^ distinguishing it from acute models (e.g., the 6-OHDA model) that often lack the progressive nature of the human disease. We selected this high-severity model specifically to evaluate the capacity of our designed nanoassemblies to decrease or reverse the upstream bioenergetic collapse of mitochondria that precedes neurodegeneration. We are also interested in the ability of lipid-peptide nanoassemblies to achieve therapeutic reprogramming of key mitochondrial, neuroinflammatory, and apoptotic gene networks. While MitoPark mice do not develop Lewy bodies, they exhibit profound retrograde axonal degeneration of dopaminergic terminals, allowing the assessment of synaptic preservation.

Despite the mechanistic suitability of the MitoPark model, effective pharmacological intervention remains a serious challenge because of two major biological barriers: the blood‒brain barrier (BBB), which restricts the passage of systemic therapeutics into the central nervous system (CNS), and the complex double-membrane structure of mitochondria, which limits intracellular drug access. The literature on nanoparticulate therapeutics evaluated in the MitoPark mouse model is quite scarce. Previous research on the MitoPark model has focused primarily on systemically administered small molecules. For example, the orally active, mitochondrion-targeted antioxidant mito-apocynin has been shown to inhibit oxidative stress and preserve nigrostriatal integrity.^10^ A targeted mito-metformin derivative has been demonstrated to have protective effects via PKD1 signaling.^11^ While these studies have validated mitochondrial targeting as a therapeutic concept, they rely on systemic distribution, which often requires high drug dosages and risks of off-target effects. Thus, a critical gap remains in the development of advanced delivery systems capable of noninvasive transport of multidrug payloads directly to the brain, leading to high local concentrations at the site of neurodegeneration.

**Fig. 1:**
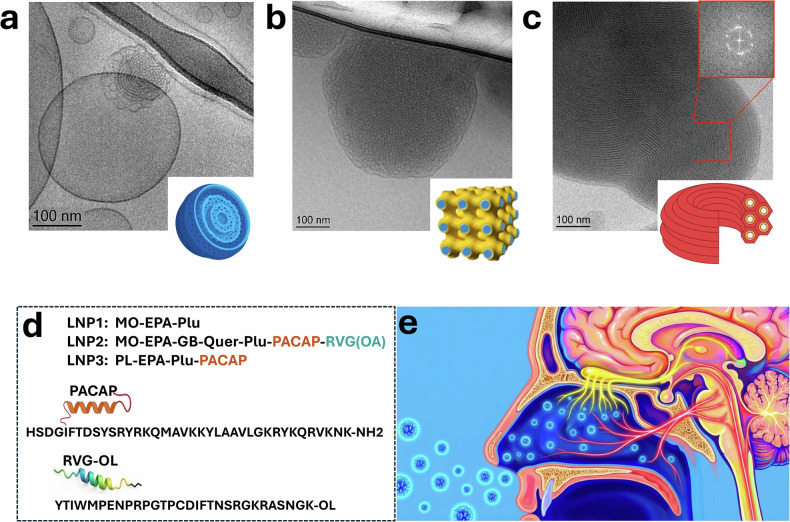
Nanostructural topologies and multicomponent compositions of liquid crystalline lipid nanoparticles (LNPs) designed for noninvasive nose-to-brain therapeutic delivery. (**a–c**) Cryo-TEM images showing the shapes and internal liquid crystalline organization of (**a**) lipid vesicular structures alongside LNPs with inner nonlamellar domains; (**b**) lipid cubosome–liquid crystalline LNPs with a cubic lipid phase organization presenting a highly organized internal arrangement characterized by periodically ordered cubic membrane domains; and (**c**) a hexosome LNP displaying features of a hexagonal phase, including repeating periodic patterns corresponding to tightly packed lipid cylinders. The inset fast Fourier transform (FFT) of the ordered region displays a distinct sixfold symmetry pattern, providing proof of the hexagonal lattice. All scale bars represent 100 nm. **d** Liquid crystalline lipid nanoparticles (denoted as LNP1, LNP2, and LNP3) are designed with multicomponent compositions, and (**e**) a scheme of noninvasive nose-to-brain therapeutic delivery through the olfactory and trigeminal nerve pathways that directly bypass the blood–brain barrier (BBB) is shown. In our strategy for targeting molecular pathways involved in mitochondrial bioenergetics and neuronal survival, LNP1, LNP2, or LNP3, which have different shapes and inner structural organization, incorporate bioactive lipids (PUFAs and/or plasmalogens) and coencapsulate neuroprotective antioxidants (GB and Quer). Peptide functionalization by pituitary adenylate cyclase-activating polypeptide (PACAP) and a rabies virus glycoprotein (RVG)-derived peptide-oleic acid conjugate (RVG-OL) is performed to increase the efficacy of nose-to-brain drug delivery. Figure 1e was enhanced using Gemini online tool

To bridge this gap, we propose a nose-to-brain delivery strategy utilizing multifunctional liquid crystalline lipid nanoparticles (LCNPs) (Fig. 1). It is intranasal and circumvents the BBB through the olfactory and trigeminal nerve pathways, facilitating direct drug transport to the CNS.^12^ Our study introduces a novel, rationally designed nanomedicine platform optimized for neuronal uptake and mitochondrial support: peptide-targeted liquid crystalline lipid nanoassemblies (cubosomes and hexosomes) (Fig. 1a–c). These self-assembled structures possess a high internal surface area ideal for solubilizing hydrophobic neuroprotective agents, specifically ginkgolide B (GB) and quercetin (Quer), which have poor bioavailability in their free forms. LNPs offer a promising way to enhance drug stability, solubility, and bioavailability while improving intracellular delivery and therapeutic efficacy.^13,14^ To ensure precise neuronal targeting, we employed a dual-peptide strategy: a rabies virus glycoprotein (RVG)-derived peptide to facilitate neuronal targeting and “homing” and a pituitary adenylate cyclase-activating polypeptide (PACAP) to increase penetration and stimulate neurotrophic signaling (Fig. 1d).

The purpose of the present study was to identify the molecular pathways and biological processes that can be beneficially altered by LNP treatment and evaluate the efficacy of targeted, multidrug-loaded nanoassemblies in mitigating the mitochondrial dysfunction characteristic of the MitoPark phenotype. To this end, we engineered a lipid matrix using the nonlamellar structural lipid monoolein and plasmalogens. Vinyl-ether phospholipids (plasmalogens) are structurally critical for synaptic function and are notably depleted in the brains of PD patients.^15^ By enriching lipid matrices with ω-3 polyunsaturated fatty acids (PUFAs), the liquid crystalline lipid nanoparticles (LCNPs) themselves become a bioactive therapeutic, aiding in membrane repair. In addition, we aimed to demonstrate that intranasal delivery of peptide-functionalized cubosomes and hexosomes can induce synergistic therapeutic effects by simultaneously suppressing oxidative stress, modulating neuroinflammation, and reprogramming transcriptional networks to support mitochondrial bioenergetics and halt dopaminergic degeneration. To date, no study has investigated the potential of intranasal nanomedicines to deliver multidrug payloads directly to the brain in the studied genetic MitoPark (MP) model. In our approach, we engineered nanocarriers using PUFAs and plasmalogens, which are essential for synaptic fusion that is depleted in PD, thereby transforming LNP carriers into bioactive therapeutics. By functionalizing the LNPs with RVG (for neuronal homing) and PACAP (for neuroprotection), we aimed to provide a proof-of-concept that nose-to-brain delivery can modulate mitochondrial gene expression in the MitoPark mouse brain.

## Results

### Rational design and physicochemical characterization of peptide-functionalized nanoassemblies

To overcome challenges in brain delivery and neuronal targeting, we engineered three distinct polyunsaturated fatty acid (PUFA)-enriched liquid crystalline lipid nanoparticle (LCNP) formulations. Engineered LNPs (LNP1, LNP2, and LNP3) possess specific physicochemical properties that are strategically optimized for noninvasive intranasal delivery, neuronal targeting, and multidrug encapsulation. We hypothesized that combining ginkgolide B (GB), quercetin (Quer), and ω-3 polyunsaturated fatty acids (PUFAs) within a single lipid-based nanoparticle could yield synergistic or multitargeted therapeutic benefits. Both GB and Quer are known for their antioxidant, anti-inflammatory, and mitochondrial-protective properties.^16–22^ They were coencapsulated within a lipid matrix to address the challenges of poor release and limited bioavailability in physiological environments. The rationale for combining ω-3 PUFAs (such as EPA) with plasmalogens stems from their complementary roles.^23,24^ EPA contributes not only as a structural component but also as a bioactive lipid that exerts anti-inflammatory and neuroprotective effects. The combination of EPA with plasmalogen is expected to increase lipid membrane-targeting capacity, allowing the integration of bioactives into neuronal membranes.

#### Structural characterization of cubosomal and hexosomal nanoassemblies for nose-to-brain delivery

**Fig. 2:**
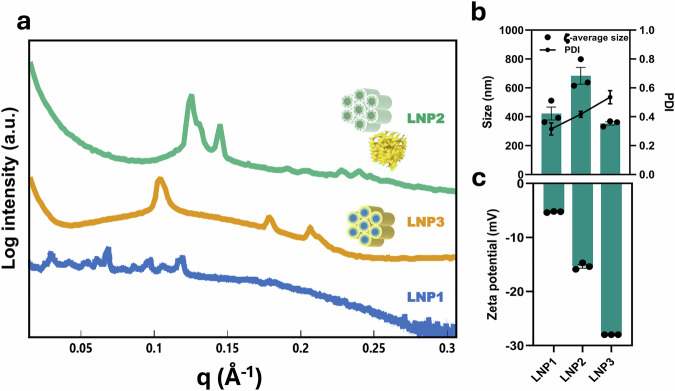
Liquid crystalline lipid nanoparticle structural and physicochemical characterization revealing the properties of the PUFA-enriched LNPs depending on the functionalization with PACAP and RVG‑OL peptides. **a** Synchrotron small-angle X-ray scattering (SAXS) patterns of monoolein-based nanoassemblies stabilized with Pluronic F127 (LNP1: MO-EPA-Plu) in excess aqueous medium and liquid crystalline nanoparticles of mixed types (hexosomes and cubosomes) formed by coencapsulation of ginkgolide B and quercetin and subsequent functionalization with PACAP and RVG-OL (LNP2: MO-EPA-Plu-GB-Quer + PACAP/RVG-OL and LNP3: PL-EPA-Plu + PACAP). Measurements were performed at 22 °C. **b**, **c** Hydrodynamic diameter, polydispersity (PDI), and zeta potential of LNP1-type PUFA-LNPs or those functionalized with PACAP and RVG-OL cell-penetrating peptides (LNP2) or PACAP (LNP3)

The internal nanostructure, which dictates therapeutic payload capacity and release, was precisely characterized using synchrotron small-angle X-ray scattering (SAXS). SAXS experiments were conducted to elucidate the multicompartment organization of the formulated therapeutic nanoassemblies. The results are presented in Fig. 2a. The SAXS scattering pattern obtained for the dispersed MO-Plu-EPA liquid crystalline lipid nanoparticles (denoted as LNP1) displayed weak Bragg peaks that could not be assigned to a distinct liquid crystalline phase. This suggested an uneven distribution of EPA in the dispersed bilayer membranes (vesicles) coexisting with small hexosomes. Structural modifications of the nanoparticles were observed following encapsulation of the hydrophobic antioxidants GB and Quer into the lipid dispersions, which induced composition-dependent nanostructural transformations of the particles (see the plot for LNP2).

The SAXS diffractogram of the mixed MO-EPA-Plu-GB-Quer (denoted as LNP2) nanoassemblies functionalized with the peptide PACAP is shown in Fig. 2a. The strong Bragg reflections at *q*-vector values of 0.125, 0.22, and 0.25 Å^−1^ (spaced at a ratio of 1:√3:√4) were indexed as Bragg peaks of an inverted hexagonal phase H_II_. The corresponding lattice parameter, a_(HII)_, was determined to be 5.80 nm. It characterizes the inner structure of the dispersed LCNPs. In addition to the diffraction peaks of the H_II_ phase, further Bragg peaks with *q*-vector positions spaced at a ratio of √6: √8: √14: √16: √20: √22 … were detected in the LNP2 dispersion. These peaks were indexed as the (211), (220), (321), (400), (420), (332), (422), and (431) reflections of a bicontinous gyroid cubic lattice of the *Ia3d* space group Q^IIG^ (Fig. 2a, green plots). A lattice parameter a_Q(*Ia3d*)_ = 12.31 nm was determined from the sequence of Bragg *q*-vector positions at 0.125, 0.144, 0.191, 0.204, and 0.228 Å^−1^. These cubic phase peaks were considerably weaker than those of the dominant H_II_ liquid crystalline phase.

On the other hand, the LCNPs containing plasmalogen lipids enriched with EPA and coupled to the PACAP peptide (LNP3: PL-EPA-Plu+PACAP) exhibited three dominant Bragg peak maxima at *q* values of 0.105, 0.179, and 0.207 Å^−1,^ with *q*-vector positions spaced at a ratio of 1:√3:√4. The formed hexosome particles, with an inverted hexagonal (H_II_) phase structure, exhibited a corresponding lattice parameter, a_(HII)_ = 7.12 nm. These results revealed a highly organized internal liquid crystalline structure of the antioxidant-loaded LNPs, a feature optimal for stably coencapsulating multiple therapeutic agents with different chemical properties.

The hydrodynamic sizes of the investigated LNPs were measured and revealed a narrow size distribution (Fig. 2b). The determined mean hydrodynamic diameters correlated with reproducible biodistribution properties. The surface of the liquid crystalline lipid nanoparticles was functionalized with a dual-peptide system: a PACAP peptide to initiate neurotrophic signaling at the target cell directly and a cell-penetrating RVG-OL peptide to act as a “homing peptide”, ensuring enhanced uptake into neuronal cells. The zeta potentials of all the studied LCNPs formulated with either PACAP or RVG-OL were determined in NaCl (1×10 − 3 M) medium, and the results are summarized in the histograms in Fig. 2c. LNP1 exhibited a slightly negative ζ-potential of approximately −5.28 ± 0.11 mV. In contrast, LNP2 had a ζ-potential value of –15.33 ± 0.60 mV, which was less negative than the value (–32 mV) before its complexation with the positively charged peptides PACAP and RVG-OL. LNP3 displayed a negative surface charge with a ζ-potential of approximately −28 mV, reflecting a shift from −52 mV to −28 mV after functionalization with the cationic peptide PACAP. These results indicate that dual surface functionalization with the PACAP and RVG‑OL peptides moderate the ζ‑potential to −15.33 mV, yielding a moderately negative surface charge. However, for LCNPs, membrane and cellular interactions depend not only on surface charge but also on lipid composition, internal nanostructure, and phase behavior.^25^ The resulting physicochemical properties are expected to promote more efficient mucus penetration and cellular uptake while maintaining sufficient stability to minimize aggregation in the nasal environment.^26^

### Internalization of LNP formulations in differentiated SH-SY5Y cells and activation of pro-survival signaling pathways in vitro

**Fig. 3:**
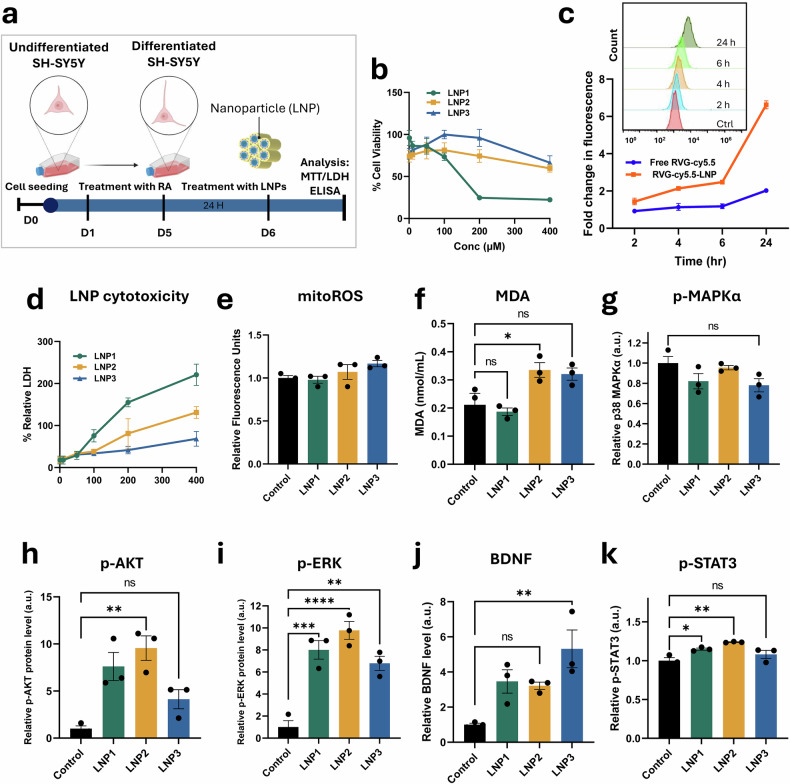
In vitro cellular studies of liquid crystalline lipid nanoparticles in differentiated SH-SY5Y cells, validating the fundamental design principles of the LNPs. **a** Experimental treatment scheme of SH-SY5Y cells after differentiation with retinoic acid (RA) for 5 days and incubation with LNP formulations (LNP1, LNP2, LNP3) for 24 h in FBS-free medium. This treatment was followed by assays for viability, cytotoxicity, mitochondrial oxidative stress, and signaling pathway activation. **b** Cell viability (MTT assay) of LNPs in a dose-dependent manner (1–400 μM). **c** Cellular uptake kinetics of RVG-Cy5.5–conjugated LNPs *vs* free Cy5.5 dye measured by flow cytometry, showing enhanced, time-dependent uptake of RVG-Cy5.5–LNPs. **d** cytotoxicity (LDH release) of LNPs (1–400 μM). **e**–**k** In vitro assessment of oxidative stress markers (MDA) and mitochondrial oxidative stress markers (mitochondrial ROS detected by MitoSOX Red) and survival/neurotrophic signaling markers (p-AKT, p-ERK, p-STAT3 Tyr705, p-MAPKα, and BDNF) following LNP treatment. The data are reported as the mean **±** SD (*n* = 3; *p < 0.05, **p < 0.01, ***p < 0.001, ****p < 0.0001)

To assess the internalization of the LNP formulations and their ability to activate pro‑survival signaling pathways, we used human SH‑SY5Y neuroblastoma cells differentiated with retinoic acid (RA). Neuronally derived cells were exposed to three formulations (LNP1, LNP2, and LNP3) for 24 h (Fig. 3a). The results of cell viability assays revealed a dose-dependent reduction in cell survival across all LNP-treated groups. In particular, compared with LNP2 and LNP3, LNP1 exhibited greater cellular toxicity at a dose of ≥200 μM (Fig. 3b), which was further supported by the results of the cytotoxicity (LDH) analysis (Fig. 3d). Hence, the superior cytocompatibility of LNP2 and LNP3 was established at therapeutic concentrations (Fig. 3b, d).

The cellular uptake of the RVG-Cy5.5-conjugated LCNPs was investigated in comparison with that of the control free Cy5.5 dye using flow cytometry. Fluorescence emission analysis revealed a time-dependent accumulation of RVG-Cy5.5-conjugated LNP in SH-SY5Y cells, with markedly increased uptake after 24 h compared with that of the free dye (Fig. 3c). The uptake kinetics of RVG-Cy5.5-conjugated LNPs steadily increased during a 2 to 24 h incubation period, with significant uptake occurring from 6 and 24 h. In contrast, free Cy5.5 dye showed a slower uptake profile over longer exposure times. The flow cytometry data indicated that the RVG-Cy5.5-LNPs were internalized by cells in a time-dependent manner, with extended incubation enhancing uptake efficiency. Such kinetics may be favorable for ensuring the sustained intracellular release of therapeutic agents.^27–30^ The results in Fig. 3c provide clear, quantitative evidence for the effectiveness of our active targeting strategy.

Oxidative stress markers did not markedly alter mitochondrial superoxide (mitoROS) levels following LNP exposure (Fig. 3e). However, the levels of malondialdehyde (MDA), a marker of lipid peroxidation, increased in cells treated with LNP2 compared with those in untreated control cells (Fig. 3f). Subsequent experiments revealed that the modest lipid oxidation effect of LNP2 and LNP3 did not lead to long-term neuroinflammation in vivo.

Once internalized, the ability of the LNPs to modulate key intracellular signaling pathways was assessed. Inside the cells, the LNPs initiated their therapeutic action by activating a cascade of key neuroprotective signaling pathways. This included robust phosphorylation of AKT and ERK, which are central mediators of cell survival and plasticity, as well as activation of the neuroprotective transcription factor STAT3. Moreover, plasmalogen-based LNP3 nanoformulation specifically upregulated the expression of the neurotrophic factor BDNF (Fig. 3g–k).

To investigate the effects of LNPs on neurotrophic and survival signaling pathways, SH-SY5Y cells were treated with LNPs and analyzed via ELISA. The data presented in Fig. 3g revealed no significant effect on p38 MAPK (p-MAPKα) modulation (Fig. 3g). However, AKT and STAT3 phosphorylation (p-STAT3 Tyr705) was upregulated in LNP1- and LNP2-treated cells (Fig. 3h, k). Moreover, extracellular signal-regulated kinase (ERK) protein levels were consistently elevated in all LNP groups. Notable differences in BDNF expression were observed between the LNP3-treated group and the control group, supporting neuronal survival and repair (Fig. 3i, j).

These key in vitro findings provide a cellular foundation for the effects observed in vivo, demonstrating that the rationally designed LCNPs directly enable efficient cellular uptake and activation of the pro‑survival mechanisms necessary for a meaningful therapeutic outcome.

### Intranasal LNP administration and in vivo efficacy of LNP therapy in a mechanistic model of Parkinson’s disease

**Fig. 4:**
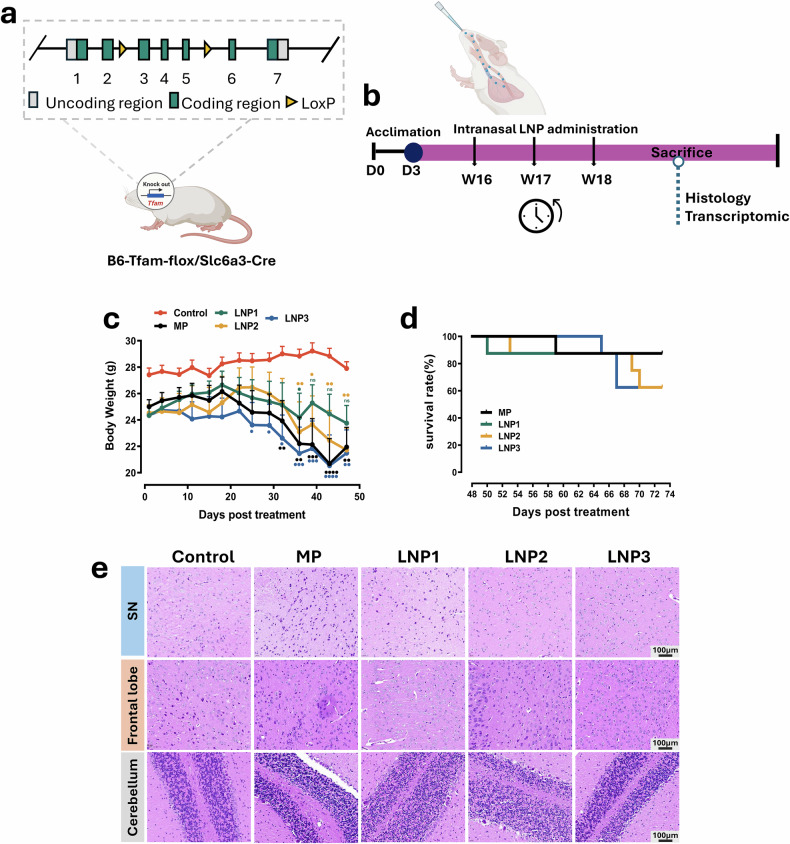
In vivo survival data (body weight and Kaplan‒Meier) and histology (H&E) results for the therapeutic PUFA-enriched lipid nanoassemblies (LNP1, LNP2, and LNP3). The survival and body weight changes in the B6-Tfam-flox/Slc6a3-Cre-MP mouse model (MitoPark) after intranasal LNP treatment. **a** The MitoPark model (MP) was generated by crossing B6-Tfam-flox mice with Slc6a3-Cre drivers, resulting in targeted deletion of mitochondrial transcription factor A (*Tfam*) in dopaminergic neurons.^7,8,60^ **a**, **b** Schematic representation of the conditional knockout strategy targeting *Tfam* in dopaminergic neurons and the experimental workflow, including intranasal administration of LNP formulations (LNP1, LNP2, LNP3). **c**, **d** Body weight measurements and Kaplan‒Meier survival analysis of WT control, MP (MitoPark disease state), and LNP-treated MP mice showing progressive weight loss, accompanied by reduced survival compared with that of control mice. The results are reported as the mean ± SD (*n* = 8; *p < 0.05, **p < 0.01, ***p < 0.001, ****p < 0.0001). Statistical differences were determined using two-way ANOVA followed by Dunnett’s multiple comparisons test. **e** H&E staining of frontal lobe, cerebellum, and substantia nigra (SN) tissues from the B6-Tfam-flox/Slc6a3-Cre MP mouse model showing histopathological alterations in MP mice compared with those in control and LNP-treated mice (scale bar is 100 μm)

To address the urgent need for disease-modifying therapy in a MitoPark (MP) mouse model, we evaluated the in vivo efficacy of three distinct liquid crystalline lipid nanoparticle (LCNP) formulations administered noninvasively via the intranasal route. The mechanistically faithful and rigorously validated MitoPark conditional knockout mouse model electively ablated mitochondrial transcription factor A (*Tfam*) in dopaminergic neurons, thereby recapitulating the primary mitochondrial dysfunction that underlies the pathogenesis of Parkinson’s disease (PD) (Fig. 4a). At 16 weeks of age, when disease pathology was well established, MitoPark mice received intranasal LNP formulations. To determine the duration of LNP treatment of three weeks, we considered the time-dependent body weight loss in diseased animals compared with that in controls (Fig. 4c). The choice of a three‑week regimen was justified by the overall trend of progressive body weight reduction, which reflects the severity of the disease state in the studied MP model.

Following a 3-day acclimation period (D0–D3), the mice received intranasal administration of PBS (for wild-type [WT] controls and the MP model) or one of the three LNP formulations (LNP1, LNP2, or LNP3) (Supplementary Table 1) beginning at week 16 for 3 weeks, after which they were subjected to behavioral testing and endpoint analyses. Since PD is characterized by motor dysfunction and cognitive dysfunction, we recorded the behavior of MP mice before and after treatment with LNPs using the rotarod test and buried food-seeking test (Supplementary Fig. 2).

#### Survival outcomes following intranasal LNP therapy in the MitoPark model

Three-week intranasal treatment of the MitoPark mouse model started when disease pathology fully developed at week 16 (Fig. 4b). Systemic assessment revealed measurable treatment-related effects. While untreated MP mice exhibited a progressive and severe decline in body weight, a hallmark of advancing neurodegeneration, mice treated with LNP formulations (particularly LNP1 and LNP2) showed significant attenuation of body weight loss during the mid-treatment phase (Fig. 4c).

Survival analysis performed over the observation period did not reveal a clear improvement in lifespan in LNP-treated mice compared with that in untreated MP controls (Fig. 4d).

With respect to the treatment doses used, the behavioral assessment did not reveal statistically significant improvements in either motor or olfactory function following treatment. In the rotarod test, untreated MP mice exhibited persistently low latencies to fall throughout the study period (Supplementary Fig. 2a, b). Similarly, performance in the olfactory-dependent buried food test did not differ significantly among the experimental groups (Supplementary Fig. 2c, d). The absence of detectable behavioral effects in this first-of-its-kind study may be attributable to the relatively small cohort size, the low treatment doses, and the advanced disease stage at which treatment was initiated in the MitoPark model.

#### Neuroprotective effects of the LNPs and neuropathological safety by H&E imaging

The recovery effects after LNP administration were directly supported by extensive histological evidence of profound neuroprotection at both the cellular and tissue levels (Fig. 4e). Hematoxylin and eosin (H&E) staining was used to examine tissue structure, cellular morphology, and pathological changes such as neuronal degeneration. Untreated MP mice displayed classic and severe signs of degeneration, including a disorganized and rarefied cellular architecture, widespread neuronal loss with pyknotic nuclei, and spongiform changes in the substantia nigra (SN), frontal lobe, and cerebellum (Fig. 4e). These pathological hallmarks were clearly and dramatically attenuated in animals treated with the LNP2 and LNP3 formulations, which exhibited restored tissue cytoarchitecture and healthier neuronal morphology (Fig. 4e). These histological results complement previously reported 21-day safety evaluations of the liquid crystalline lipid formulation in C57BL/6JGpt mice.^31^

#### Analysis of the expression of tyrosine hydroxylase (TH) and ATP5A1 as neuroprotective markers

**Fig. 5:**
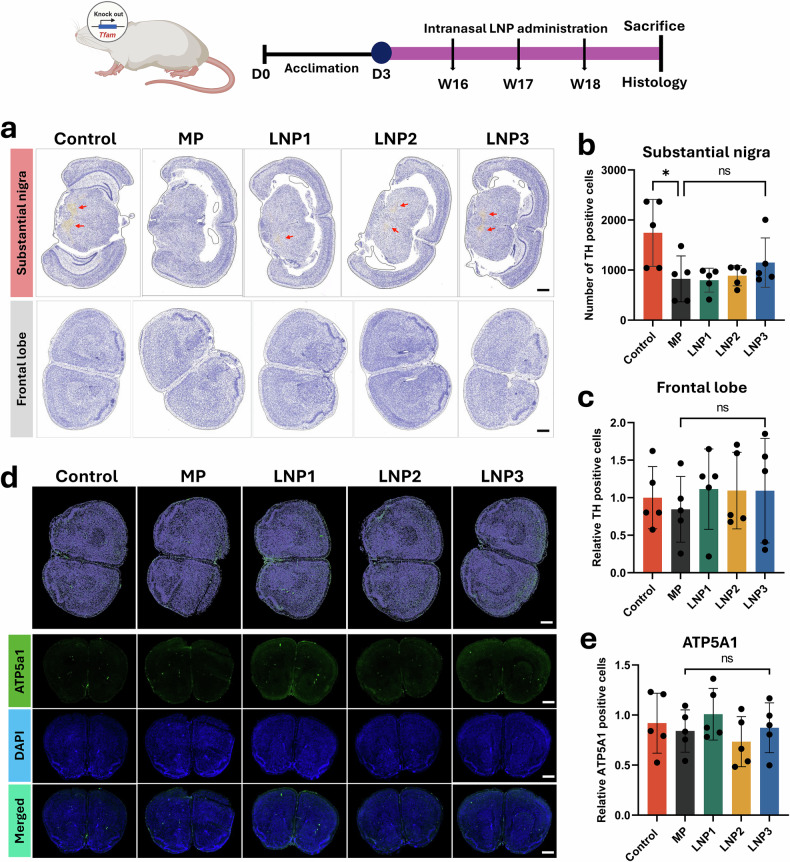
In vivo specific neuroprotective staining for dopaminergic neurons (TH) and mitochondrial integrity (ATP5A1). **a**–**c** Tyrosine hydroxylase (TH) immunostaining of the substantia nigra and frontal lobe. MP mice displayed a slight reduction in TH-positive cells (red arrows) in the SN, whereas the LNP2 and LNP3 groups exhibited partial modulation (**b**), but no changes were detected in the frontal lobe (**c**). **d** Immunofluorescence staining of ATP5A1 (green) with DAPI nuclear counterstain (blue) in control, MP, and LNP-treated mouse brains. **e** The number of ATP5A1-positive cells did not decrease in MP mice, but the number of LNP1-positive cells was slightly restored. (Scale bar is 1000 μm.) The results are reported as the mean ± SD (*n* = 5; *ns* > 0.05; *p < 0.05). Statistical analysis was performed using one-way ANOVA followed by Dunnett’s multiple comparisons test

The neuroprotective effects of LNPs on key pathological cell types and organelles in the MitoPark model were evaluated by assessing mitochondrial integrity and dopaminergic neuronal survival following intranasal administration. For this purpose, brain tissues were stained for markers of mitochondrial integrity (ATP5A1) and dopaminergic survival (TH) (Fig. 5a–e). TH immunostaining revealed a significant reduction in TH-positive cells in the substantia nigra of MitoPark mice compared with controls (*p* = 0.0123; Fig. 5a, b). Compared with no treatment, treatment with LNP2 or LNP3 was associated with moderate preservation of TH-positive cells. However, no statistically significant differences were observed (*p* = 0.9981 and *p* = 0.6642, respectively). Quantification of ATP5A1 immunostaining revealed no statistically significant differences between MitoPark mice and controls (Fig. 5d, e; *p* = 0.9668), whereas a modest increase was observed in response to LNP1 treatment (*p* = 0.6871). Therefore, no clear trend in ATP5A1 expression was detected under the examined treatment doses.

These findings confirm that mitochondrial dysfunction contributes to dopaminergic vulnerability, which is consistent with the neurodegenerative phenotype observed by H&E staining. The protective effects of LNP2 and LNP3 on neuronal integrity align with the transcriptomic data showing enrichment of mitochondrial and neuronal survival pathways (see the results presented in the next sections). Taken together, these multilevel data indicate that intranasal LNP therapy may help preserve the structural, cellular, and functional integrity of the nigrostriatal pathway, thereby mitigating disease pathology.

### Intranasal LNP treatment induces broad therapeutic reprogramming of core mitochondrial, neuroinflammatory, and apoptotic gene networks

#### Differential gene expression and clustering analysis of MP mice treated with LNP formulations

To elucidate the molecular mechanisms underlying the established in vivo efficacy of intranasal LNP therapy, we conducted a comprehensive transcriptomic analysis of hippocampal brain tissue. This region is critically involved in learning and memory and is particularly vulnerable to neurodegenerative diseases. Although the primary pathology of the MitoPark model involves degeneration of the nigrostriatal dopaminergic system, the hippocampus is also affected in Parkinson’s disease and has been implicated in mitochondrial dysfunction, neuroinflammation, and cognitive impairment associated with disease progression. Therefore, hippocampal transcriptomic profiling was used to capture broader neurodegenerative and inflammatory responses associated with treatment.

**Fig. 6:**
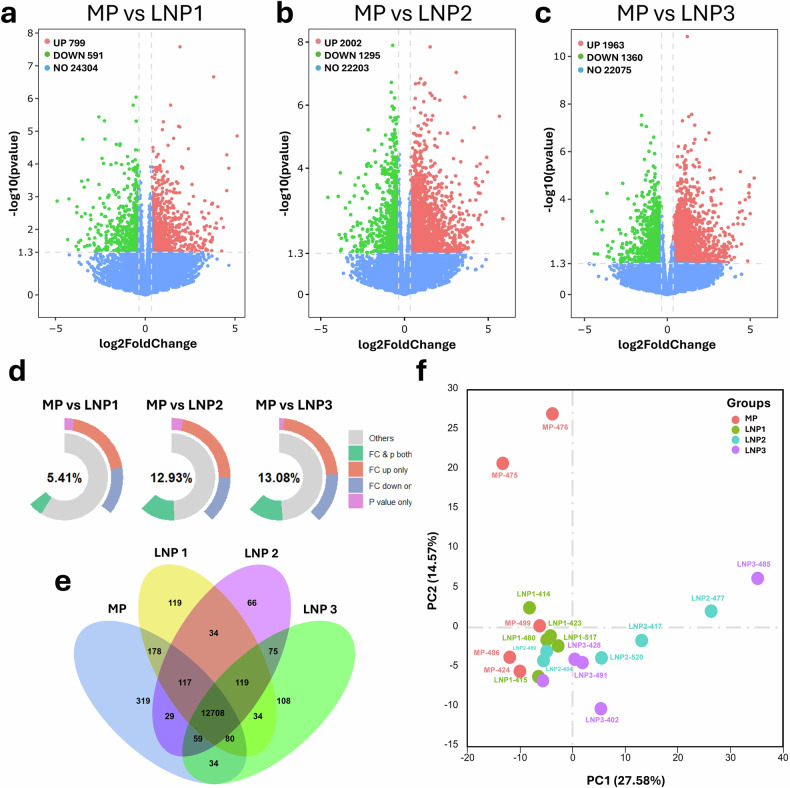
Transcriptomic analysis of the effects of PUFA-enriched LNPs in MP-treated mice (volcano plots, donut charts, Venn diagram, and PCA), revealing the large-scale genetic reprogramming underlying LNP therapeutic outcomes. Gene expression correlation and differential gene expression analysis of (**a**–**c**) volcano plots for MP *vs* LNP1, MP *vs* LNP2, and MP *vs* LNP3. Up- (red) or downregulated (green) genes are indicated by a log2FC ≥ 0.35 and *p* < 0.05. **d** Donut charts summarizing, for each comparison, the fraction of genes that passed both criteria (center percentage). The ring segments indicate the categories (FC & *p* both, FC only, *p* only, and others that do not pass the threshold). **e** Venn diagram showing the overlap of DEGs among the MP, LNP1, LNP2, and LNP3 comparisons. **f** PCA of variance-stabilized expression values. The samples were clustered by group, with PC1 (27.6%) and PC2 (14.6%). *n* = 5 per group; RNA samples were derived from hippocampal tissue

The results revealed that our LNP platform not only treats symptoms but also induces profound reprogramming of the gene expression landscape, which transforms the cellular environment from a pathological state to a restorative state. The therapeutic potency of each formulation directly correlated with the extent of transcriptomic modulation. The peptide-functionalized LNP2 and LNP3 formulations altered the expression of 3297 and 3323 genes, respectively. This represents far more extensive reprogramming than the 1390 genes altered by the pure lipid LNP1 formulation (Fig. 6a–d).

Differential expression analysis was performed between the MP group and nanoparticle-treated groups (LNP1, LNP2, and LNP3). Using a cutoff of *p* < 0.05 and a log2(fold change) ≥0.35, substantial numbers of differentially expressed genes (DEGs) were identified in each comparison. Specifically, the MP *vs* LNP1 comparison yielded 1390 DEGs, including 799 upregulated and 591 downregulated transcripts (Fig. 6a). The MP vs LNP2 comparison revealed a more profound transcriptional shift, with 3297 DEGs (2002 upregulated and 1295 downregulated; Fig. 6b). Similarly, the MP vs LNP3 comparison revealed 3323 DEGs (1963 upregulated and 1360 downregulated; Fig. 6c). The increasing DEG proportions from LNP1 to LNP3 correlate with optimized LNP compositions, which demonstrate the enhanced efficacy of LNP2 and LNP3, effectively targeting pathways relevant to PD pathology.

In addition, the donut plots in Fig. 6d summarize the proportion of genes meeting the differential expression criteria. Specifically, 5.41%, 12.93%, and 13.08% of the genes analyzed in the RNA-seq dataset were identified as DEGs in the MP vs LNP1, MP vs LNP2, and MP vs LNP3 comparisons, respectively.

A complementary Venn diagram further highlights a substantial overlap among the three treatment groups, with 12,708 genes detected as commonly expressed transcripts (Fig. 6e). Among these genes, 5.41%, 12.93%, and 13.08% met the criteria for differential expression in the MP vs LNP1, MP vs LNP2, and MP vs LNP3 comparisons, respectively (Fig. 6d). Moreover, Venn diagram analysis revealed extensive overlap in the genetic programs modulated by LNP2 and LNP3, suggesting that these genes converge on key therapeutic pathways while also possessing unique molecular footprints (Fig. 6e).

Principal component analysis (PCA) revealed partial separation of the transcriptomic profiles among the treatment groups relative to those of the MP controls (Fig. 6f). PC1 and PC2 accounted for 27.58% and 14.57% of the variance, respectively, and 42.15% of the variance, with the MP samples showing partial dispersion (MP-475 and MP-476 diverging along PC2), while the others clustered centrally. LNP1-treated samples overlapped with central MP samples, suggesting modest transcriptional shifts. In contrast, LNP2 samples extended along the positive PC1 axis and clustered quite clearly from MP. The LNP3 samples dispersed along the negative PC2 axis, reflecting distinct and heterogeneous transcriptomic responses (Fig. 6f). These results indicate differential degrees of transcriptional modulation across LNP formulations rather than uniform normalization or reversal of the MP-associated transcriptomic profile.

#### Transcriptomic profiling reveals differential gene expression patterns in pathways of neuroinflammation, apoptosis, PD pathology, and mitochondrial function

Detailed pathway enrichment analysis provided critical mechanistic insights, revealing that the LNPs broadly modulate core pathological pathways in Parkinson’s disease (PD). Specifically, LNP2 and LNP3 treatment led to the significant and coordinated downregulation of a network of pathogenic genes.

**Fig. 7:**
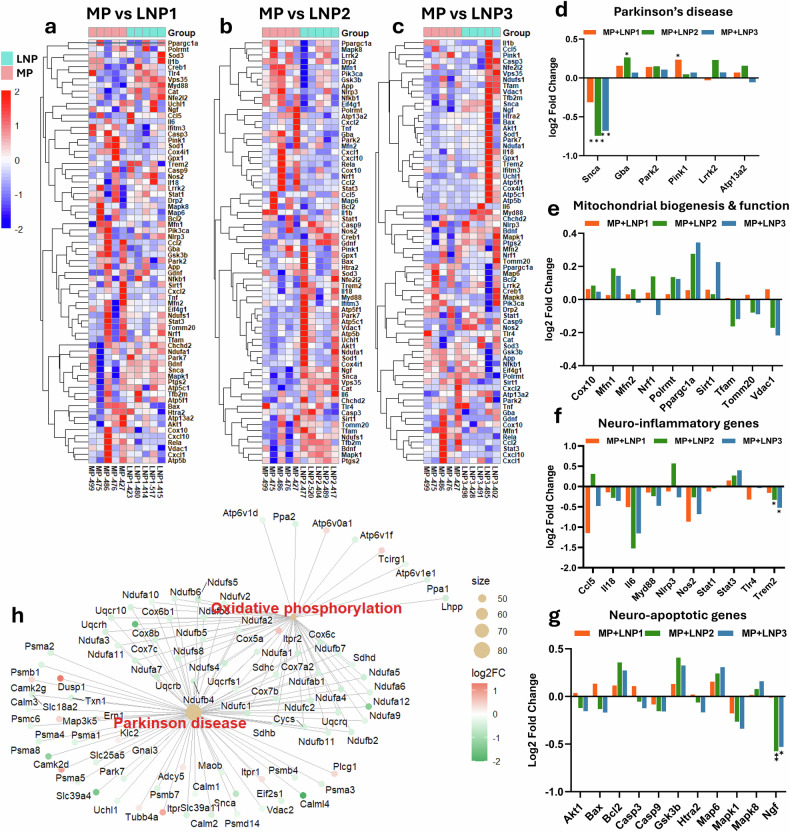
Transcriptomic remodeling in the MP model following LNP treatment, highlighting modulated pathological and neuroprotective gene sets. **a**–**c** Heatmaps of genes related to PD pathology, mitochondrial function/oxidative stress, neuroinflammation, and apoptosis for MP vs LNP1 (**a**), MP *vs* LNP2 (**b**), and MP *vs* LNP3 (**c**). **d**–**g** Log2FoldChange (MP vs LNP1/2/3) bar charts for representative gene panels. **d** PD-associated genes, **e** mitochondrial biogenesis/function, **f** neuroinflammatory mediators, and **g** neuro-apoptotic regulators. Positive bars indicate relative upregulation in MP vs LNP, and negative bars indicate suppression with LNP treatment. Statistical significance was assessed using differential expression analysis in DESeq2. The data were plotted with GraphPad Prism 9. Data are presented as the log2-fold change relative to the control (*n* = 5; *p < 0.05, **p < 0.01, and ***p < 0.001). **h** Category gene network highlighting oxidative phosphorylation and Parkinson’s disease KEGG pathways. Nodes are genes (colored by Log2FC; red = up, green = down) connected to pathway nodes (size and gene count). Differentially expressed genes were tested for KEGG with *p*adj ≤ 0.25 and FDR (p.adjust) ≤ 0.04. Term-to-term similarity was computed with pairwise terms, and edges were retained when the similarity was ≥ 0.20; the network was drawn with an emap plot (Fruchterman-Reingold layout). *n* = 5 per group; RNA samples were derived from hippocampal tissue

We constructed clustered heatmaps of selected genes relevant to PD pathology, mitochondrial function, oxidative stress, apoptosis, and neuroinflammation (Fig. 7a–c). Hierarchical clustering revealed that the MP and LNP samples grouped separately, with LNP2 and LNP3 exerting broader effects on transcriptional reprogramming than LNP1 does. Pathway enrichment analysis of the DEGs revealed significant alterations in gene expression, which are presented in bar charts as log2-fold changes in the expression of genes related to the neuroinflammatory, neuro-apoptotic, PD specific, and mitochondrial biogenesis pathways (Fig. 7d–g). Genes associated with PD pathology, such as *Snca* (α-synuclein), were significantly downregulated in the LNP2- and LNP3-treated groups. Other genes, such as *Gba, Park2, Lrrk2*, *Pink1*, *and Atp13a2*, were slightly upregulated across treatments, except for LNP1 and LNP3, whose expression modestly decreased. The expression of master regulators of neuroinflammation, including *Il6*, *Myd88*, *Nos2*, and *Trem2*, and key apoptotic effectors, such as the executioner caspases *Casp3* and *Casp9*, was strongly altered (Figs. 7d, 5f, g).

Concurrently, the LNP formulations promoted the expression of pivotal genes required for cellular machinery repair. These include the master regulator of mitochondrial biogenesis (*Ppargc1a*), essential components of the electron transport chain (such as *Cox10* and *Mfn1*), and key pro-survival factors that directly counteract apoptosis (*Bcl2*) and support mitochondrial quality control (*Pink1*) (Fig. 7e, g). As shown in Fig. 7e, mitochondrial biogenesis and functional genes were upregulated in the LNP-treated groups, with the expression of protective factors such as *Ppargc1a* markedly increased, whereas *Cox10* and *Mfn1* expression modestly increased across all the groups. In contrast, the expression of markers of mitochondrial dysfunction, such as *Tomm20* and *Vdac1*, was moderately suppressed in the LNP2 and LNP3 groups.

Neuroinflammatory gene analysis (Fig. 7f) revealed that LNP treatment markedly reduced the expression of proinflammatory mediators (*Il6* and *Il18*) and further suppressed *Myd88*, *Nos2*, and *Trem2* expression, whereas the expression of genes such as *Stat1* changed minimally. With respect to the neuroapoptotic genes (Fig. 7g), LNP2 and LNP3 moderately downregulated key effectors, particularly *Casp3* and *Casp9*. Both treatments also reduced *Bax* and *Akt* expression to an intermediate degree, while the modest upregulation of *Bcl2, Map6*, and *Gsk3β* suggested a shift toward a pro-survival state. With respect to pathway-specific effects, the modest downregulation of the expression of neuroinflammatory genes by LNP2 and LNP3 highlights their anti-inflammatory properties, likely via the suppression of NF-κB signaling (*Myd88 and Nos2*). These findings align with those of previous studies demonstrating that LNP-delivered anti-inflammatory agents attenuate microglial activation in rodent models of PD, an effect associated with improved behavioral outcomes. Similarly, the antiapoptotic effects, particularly those of LNP3, involve the modulation of the Bcl-2 family (*Bax and Casp3*), suggesting a mechanism to counteract the loss of dopaminergic neurons.^32,33^ LNP1 primarily affected circadian rhythm, Wnt signaling, and p53 signaling pathways; LNP2 strongly affected oxidative phosphorylation and Parkinson’s disease pathways; and LNP3 was enriched for ROS, apoptosis, neurodegeneration, circadian, and endocannabinoid signaling (Fig. 7h and Supplementary Fig. 5a, b).

#### Molecular insights into MP and liquid crystalline lipid nanoparticle interactions: KEGG and GO enrichment analyses reveal LNPs as modulators of mitochondrial and neurodegenerative signaling

**Fig. 8:**
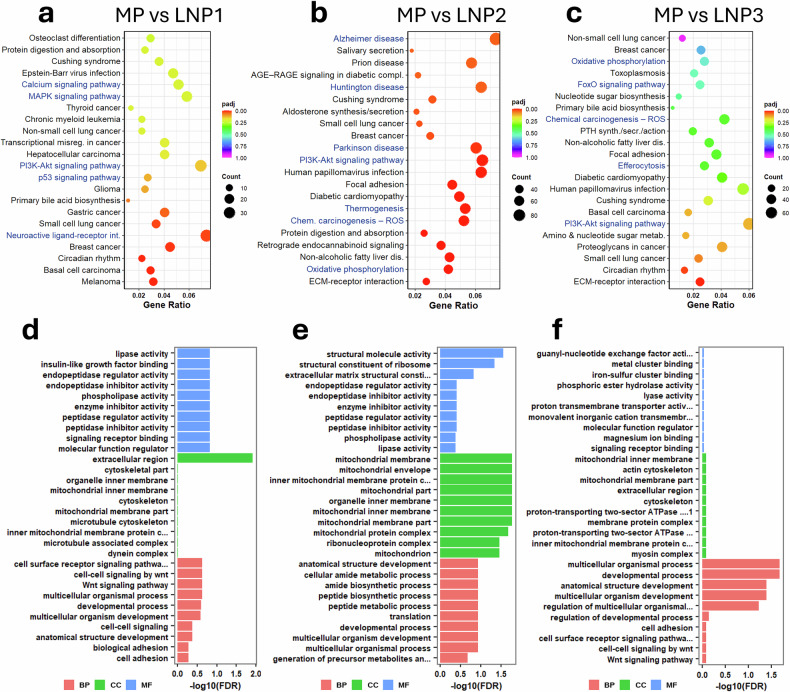
High-level pathway analysis of genes whose expression differed between the MP-treated group and the LNP-treated group: KEGG, Gene Ontology (GO), and GSEA enrichment bubble and bar plots categorizing functional reprogramming. **a**–**c** KEGG bubble plots showing enriched pathways for MP vs LNP1, MP vs LNP2, and MP vs LNP3. Node size represents the gene count, and the color scale indicates the adjusted *p* value (*p*adj ≤ 0.05). **d**–**f** GO enrichment analysis results categorized into molecular function (MF, blue), cellular component (CC, green), and biological process (BP, red) categories. *n* = 5 per group; RNA samples were derived from hippocampal tissue. Adjusted *p* values (*p*adj) were calculated using Benjamini–Hochberg false discovery rate (FDR) correction

High-level pathway analysis integrates individual gene expression changes into a coherent biological framework. KEGG pathway enrichment revealed that while LNP1 primarily modulated general stress-related pathways, LNP2 strongly enriched mitochondrial function, neurodegenerative disease mechanisms, and ECM-receptor interaction pathways, which are central components of Parkinson’s disease pathology. LNP3 was characterized by broader effects on oxidative stress, apoptosis, and cytoskeletal modules (Fig. 8).

Kyoto Encyclopedia of Genes and Genomes (KEGG) pathway analysis (Fig. 8a-c and Supplementary Fig. 6a-f) revealed that the expression of genes related to the signaling pathways of IL-17, MAPK, TNF, apoptosis, p53, and NF-κB was upregulated in the MP *vs* LNP1 comparison, which reflects the activation of stress and survival signaling cascades. The downregulated pathways were enriched for nucleotide metabolism, thiamine/pyrimidine metabolism, PI3K-Akt, and the biosynthesis of cofactors (Fig. 8a and Supplementary Fig. 6a, b), which is consistent with the suppression of maladaptive metabolic reprogramming in PD.^34^

With respect to the MP *vs* LNP2 comparison, KEGG analysis revealed upregulated terms, including ECM-receptor interaction, focal adhesion, PI3K-Akt, and AGE-RAGE signaling, which refer to extracellular and stress-response signaling (Fig. 8b and Supplementary Fig. 6c, d). Notably, the downregulated pathways included oxidative phosphorylation, Parkinson’s disease, Huntington’s disease, Alzheimer’s disease pathways, ribosome, proteasome, and thermogenesis, which are robust biomarkers that target mitochondrial dysfunction and protein homeostasis.

With respect to the MP *vs* LNP3 comparison, KEGG analysis revealed upregulation of the PI3K-Akt, MAPK, FoxO, TGF-β, and apoptosis pathways, in addition to ECM-related processes (Supplementary Fig. 6e). This confirms the multipathway-driven effect of LNP3 (regulation of signaling and structural remodeling), as shown by the GO terms (Fig. 8f). The downregulated pathways included oxidative phosphorylation, Parkinson’s disease, Alzheimer’s disease, Huntington’s disease, thermogenesis, ribosome, and carbon metabolism, indicating the suppression of neurodegenerative and metabolic stress pathways (Supplementary Fig. 6f).

We further identified genes that were differentially expressed between the MP- and LNP-treated groups and subjected them to pathway enrichment analysis. Through the analysis of annotated genes, we derived primary Gene Ontology (GO) terms such as ‘molecular function’ (MF), ‘cellular component’ (CC), and ‘biological process’ (BP). GO enrichment revealed both unique and shared functional pathways across LNP1, LNP2, and LNP3 treatments in MP mice (Fig. 8d–f). In the MP *vs* LNP1 comparison, the most significantly enriched MF terms included peptidase regulator activity, peptidase inhibitor activity, enzyme inhibitor activity, and phospholipase activity, indicating that LNP1 primarily affects protease regulatory networks and lipid-associated enzymatic processes. Consistent with these functions, CC enrichment highlighted the extracellular region, suggesting modulation of extracellular signaling and membrane-associated regulatory mechanisms (Fig. 8d).

In contrast, the MP vs LNP2 comparison strongly enriched mitochondria-related cellular components, including mitochondrial membrane parts, mitochondrial protein complexes, inner mitochondrial membranes, and organelle inner membranes. These CC terms were associated with BP categories such as peptide metabolic process, peptide biosynthetic process, translation, and generation of precursor metabolites and energy, indicating that LNP2 treatment is closely associated with mitochondrial organization and cellular metabolic restoration (Fig. 8e).

With respect to the MP vs LNP3 comparison, the enriched BP categories were associated mainly with multicellular organismal processes, developmental processes, and the regulation of signaling pathways. These results suggest that LNP3 influences cytoskeletal/structural signaling networks (Fig. 8f). Notably, several enriched GO categories overlapped across treatments, particularly those related to enzyme inhibitor activity, peptidase regulator activity, and structural molecule activity, highlighting shared mechanisms linked to protein regulation and cellular stability.

#### Pathway enrichment analysis and GSEA identify distinct signatures across LNP formulations

**Fig. 9:**
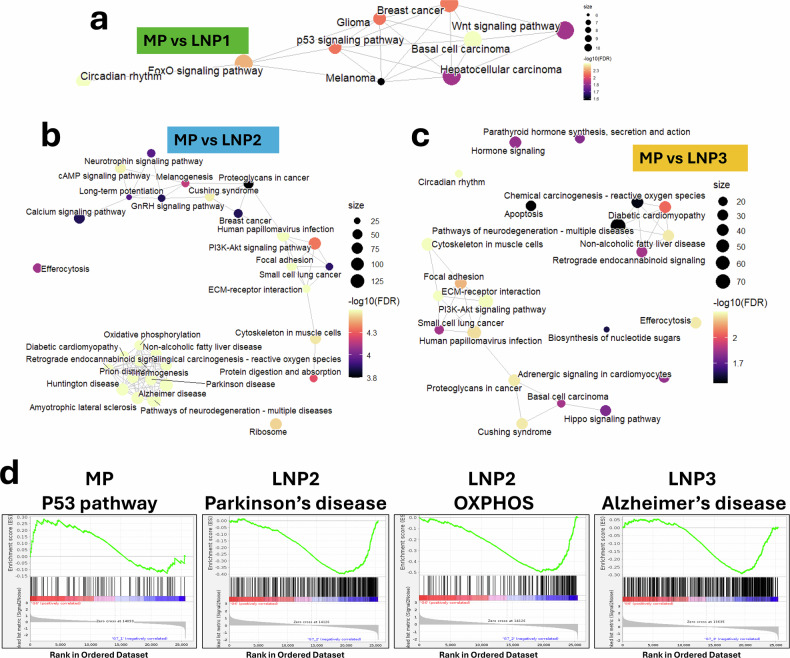
Integrated pathway‒pathway enrichment network analysis of DEGs in the MP- *versus* LNP-treated groups (KEGG network maps and GSEA plots revealing interconnected pathways and signature reversal). *Top panel*. Enrichment maps showing significantly enriched KEGG pathways for the **a** MP vs LNP1, **b** MP vs LNP2, and **c** MP vs LNP3 comparisons. Each node represents an enriched pathway, with node size proportional to the number of associated genes and color indicating statistical significance (–log10 FDR). Edges represent term-to-term similarity scores ≥ 0.20, reflecting functional relatedness. MP vs LNP1 was dominated by cancer- and stress-associated pathways (e.g., Wnt and p53 signaling), MP vs LNP2 revealed broader enrichment in mitochondrial, neurodegenerative, and ECM-receptor interaction pathways, and MP vs LNP3 was characterized by oxidative stress, apoptosis, and cytoskeletal/signaling modules. *Bottom panel*. **d** Gene set enrichment analysis (GSEA) plots highlighting significantly enriched hallmark pathways, including p53 signaling (MP baseline), Parkinson’s disease and oxidative phosphorylation (LNP2), and Alzheimer’s disease (LNP3). *n* = 5 per group; RNA samples were derived from hippocampal tissue

Gene set enrichment analysis (GSEA) plots provided conclusive evidence from KEGG enrichment network maps (Fig. 9a–d) and confirmed that MP was enriched for strongly activated stress- and apoptosis-related signaling cascades, including the p53 pathway, AMPK, and MAPK (Fig. 9d and Supplementary Fig. 7a), which is consistent with mitochondrial dysfunction and cell death as key drivers of pathology. Furthermore, the MP *vs* LNP1 comparison revealed stress-associated pathways that are not directly related to mitochondrial bioenergetics (Fig. 9a).

LNP2 treatment induced strikingly different enrichment profiles. GSEA powerfully demonstrated that LNP2 treatment specifically and significantly suppressed the ‘Parkinson’s disease’ gene signature while simultaneously restoring the critical ‘oxidative phosphorylation’ pathway, the primary energy-generating process that is severely impaired in this model (Fig. 9b, d). These compelling data show that the observed functional recovery is driven by synergistic reprogramming of the core gene networks that govern mitochondrial health, neuroinflammation, and neuronal survival.

In comparison, LNP3 treatment was associated with enrichment of broader neurodegenerative pathways, particularly the Alzheimer’s disease gene set (Fig. 9c, d). While this finding indicates a potential role for LNP3 in targeting common mechanisms underlying multiple neurodegenerative disorders, its impact on mitochondrial function appeared less pronounced than that of LNP2.

## Discussion

Traditional single-target therapies have largely failed in complex neurodegenerative diseases because of the multifactorial nature of these conditions, which require multitarget approaches. Our work shows that rationally designed, multicomponent nanoassemblies, delivered noninvasively, can counteract disease progression in a mechanistically faithful preclinical model.

Mitochondrial dysfunction is a central driver of dopaminergic neuron loss in PD. To investigate mitochondria-targeted therapies, we used the MitoPark mouse model, generated by selective ablation of mitochondrial transcription factor A (Tfam) in midbrain dopaminergic neurons via the Cre-loxP system. This model induces impaired mtDNA maintenance, severe respiratory chain deficiency, and progressive bioenergetic failure, recapitulating key features of clinical PD, including adult-onset motor and non-motor deficits.^8,35–38^

Using a conditional *Tfam* knockout (MitoPark) model of PD, we demonstrated that nose-to-brain delivery of peptide-functionalized liquid crystalline lipid nanoparticles enriched with polyunsaturated fatty acids can attenuate mitochondrial dysfunction and neuronal degeneration. By integrating antioxidants such as ginkgolide B and quercetin within cubosome/hexosome nanoassemblies and functionalizing the surface with RVG-oleic acid (RVG-OL) and PACAP peptides, we established a multitargeted nanocarrier system capable of improving mitochondrial integrity, reducing oxidative stress, and enhancing neuronal survival pathways. The innovative use of dual peptide functionalization of nanocarriers is crucial for their performance. First, the RVG-OL peptide enhanced neuronal targeting through nicotinic acetylcholine receptor interactions, a strategy similar to that employed for siRNA delivery.^35^ Here, we extended this principle by anchoring the RVG-OL conjugate to PUFA-enriched cubosomes and hexosomes, thereby achieving efficient neuronal uptake and nose-to-brain delivery. Moreover, the incorporation of PACAP in the LNPs promoted neurotrophic signaling and generated synergistic effects.

Our in vitro analyses of differentiated SH-SY5Y cells revealed that LNPs increased neuronal viability and suppressed stress-related MAPK signaling while simultaneously activating the pro-survival AKT/ERK/STAT pathway. These findings are consistent with reports on STAT3 activation and long-term neuroprotective effects against oxidative apoptosis in PD models.^36^ In another context, studies in cardiac and neuronal cells have shown that reduced STAT3 activity enhances susceptibility to oxidative injury, supporting its cytoprotective role.^37^ Moreover, mitochondrial STAT3 is essential for guarding against oxidative stress-induced apoptosis through the ASK1/p38 MAPK signaling pathway.^38^ Therefore, the simultaneous activation of ERK/STAT3 signaling may represent a compensatory mechanism to counterbalance oxidative injury, inhibit apoptosis and enhance antioxidant defenses to maintain neuronal survival. The activation of AKT/ERK pathways by LNP2, accompanied by a modest increase in brain-derived neurotrophic factor (BDNF) levels, observed across all LNP-treated groups relative to the control, reflects the neurotrophic potential of the nanoformulations.

The in vivo efficacy of our PUFA-enriched LNP formulations in the *Tfam* conditional knockout PD model represents a significant advancement in targeted noninvasive delivery for the treatment of neurodegenerative diseases. Intranasal administration bypasses the blood–brain barrier, enabling direct access to olfactory and trigeminal pathways for brain penetration, as supported by prior studies on nanoparticle-based CNS delivery.^39–41^ *Tfam* knockout-influenced olfactory dysfunction (hyposmia) is clearly an early nonmotor symptom that worsens with disease progression.^42^ The lack of improvement in the buried food test suggests that PUFA-enriched LNPs may not have effectively delivered therapeutic agents (e.g., GB and quercetin) to olfactory bulb neurons or associated pathways (Supplementary Fig. 2). Additional contributing factors could include insufficient therapeutic dosing, regional variability in blood–brain barrier permeability, or suboptimal nanoparticle uptake influenced by cell type, receptor expression, or particle size.^43–47^

From the results of the transcriptomic analysis, we identified a substantial overlap of 12,708 genes across all three comparisons and a high intersample reproducibility and a large, shared gene set across the MP and LNP groups, which may represent key therapeutic targets (Fig. 6e and Supplementary Fig. 8-10). However, the relatively low percentage of DEGs (5.41–13.08%) suggests that the treatments induce specific rather than broad transcriptomic changes, which could be advantageous for minimizing off-target effects. Furthermore, the in vivo experiments provided further validation, as LNP treatment in *Tfam* knockout mice led to partial preservation of ATP5A1 expression and a trend toward the preservation of tyrosine hydroxylase (TH)-positive dopaminergic neurons, although the behavioral improvements were not statistically significant. Moreover, improvements in survival in LNP-treated mice emphasize the translational value of nonviral strategies for addressing mitochondrial dysfunction.^48^

The strong modulation of the oxidative phosphorylation pathway by LNP2 treatment provided a direct mechanistic link between our therapeutic strategy and the core mitochondrial dysfunction that defines PD. In the MP *vs* LNP1 comparison, the enriched networks identified were primarily associated with circadian rhythm, Wnt signaling, p53 signaling, and cancer-related pathways. These pathways are driven by upregulated genes such as *Per1, Cry1, Csnk1e*, and *Psen1*, which may perturb transcriptional and cell cycle regulatory processes. Notably, the MP *vs* LNP2 comparison strongly enriched oxidative phosphorylation and Parkinson’s disease-associated pathways. Many mitochondrial complex I and IV subunits (*Ndufa, Ndufb, Ndufs, Cox* family members) were significantly altered. The expression of Parkinson’s disease-related genes (*Park7, Snca, Maob, and Uchl1*) was also altered, highlighting the close link between mitochondrial dysfunction and neurodegeneration in the LNP2 condition.

Interestingly, the MP vs LNP3 comparison revealed enrichment of pathways related to oxidative stress (ROS), apoptosis, neurodegeneration, circadian rhythm, and endocannabinoid signaling. Differential expression of key genes such as *Mapk14, Casp7, Snca, Dnajc10*, and *Calm1* highlights the activation of cell death and stress-response mechanisms. These transcriptional changes can induce pathway modulation, which is commonly linked to neuronal stress and survival, suggesting a regulatory role in balancing pro-apoptotic and compensatory mechanisms.

A limitation of this first study investigating cubosome‑ and hexosome‑based nanomedicine delivery in the MitoPark model is the combination of relatively low drug doses, a small behavioral testing cohort, and the initiation of treatment at an advanced stage of disease progression. Although molecular, transcriptomic, and histological analyses revealed modulation of mitochondrial and neuroinflammatory pathways, the current experimental design did not yield statistically significant improvements in motor or olfactory behavior. Future studies employing higher therapeutic doses, larger cohorts, earlier intervention, and extended treatment durations will be necessary to determine whether these molecular effects translate into measurable functional recovery.

In summary, we demonstrated that our LNP2 and LNP3 cubosome‑ and hexosome‑type nanoformulations can exert multiple therapeutic effects by concurrently suppressing proinflammatory and apoptotic signaling, including the downregulation of key mediators such as *Myd88*, *Il6*, and *Casp3*. In parallel, these formulations promoted mitochondrial‑associated regulatory pathways, highlighted by the upregulation of the expression of the mitochondrial biogenesis regulator *Ppargc1a* and genes involved in oxidative phosphorylation. We conclude that multifunctional PUFA-enriched, peptide-functionalized liquid crystalline lipid nanoparticles represent a promising therapeutic platform for mitigating mitochondrial dysfunction and neurodegeneration in Parkinson’s disease. Through the incorporation of ginkgolide B, quercetin, and ω-3 PUFAs into lyotropic nanoassemblies stabilized with plasmalogens and functionalized with the peptide PACAP and an RVG-derived peptide conjugate (RVG-OL), we achieved efficient intranasal delivery that promoted cellular uptake, reduced neurotoxicity, and activated pro-survival signaling pathways in vitro. In vivo, intranasal administration of LNP formulations in MitoPark mice was associated with trends toward improved mitochondrial marker levels and dopaminergic neuronal preservation. The transcriptomic results revealed that compared with LNP1, LNP2 and LNP3 exerted broader transcriptomic remodeling characterized by the suppression of PD pathological genes (e.g., *Snca*), neuroinflammatory mediators (*Il6, Nos2, Myd88*), and apoptotic effectors (*Casp3, Casp9*), as well as the upregulation of mitochondrial biogenesis and survival regulators (*Ppargc1a*, *Pink1*, *Bcl2*). Therefore, LNP2 may preferentially modulate mitochondrial-associated pathways, which may favor “precision mitochondrial therapeutics”, which are relevant to PD, whereas plasmalogen-based LNP3 appears to act as a “broad-spectrum neuro-network stabilizer”, suggesting its potential applications in diseases such as Alzheimer’s disease, where more widespread structural and signaling deficits are present.

In summary, we identified several pathway enrichment hubs associated with the modulation of extracellular signaling, mitochondrial metabolism, and oxidative phosphorylation, as well as cytoskeletal and signaling networks with broader neuroprotective effects. These genes were enriched in the oxidative phosphorylation pathway, Parkinson’s disease, Alzheimer’s disease, Huntington’s disease, extracellular signaling programs (ECM-receptor interaction, PI3K-Akt, focal adhesion), and structural/cytoskeletal organization (ribosome, myosin complex) pathways. Our findings demonstrate the therapeutic potential of peptide-functionalized PUFA-LNPs as versatile carriers capable of modulating key pathological processes in Parkinson’s disease, including mitochondrial dysfunction, oxidative stress, apoptosis, and neuroinflammation. These results provide a foundation for the future development of intranasal nanocarrier systems as disease-modifying interventions in PD and other neurodegenerative disorders.

## Experimental section

### Materials

The plasmalogen lipid was obtained from our previous work,^15^ whereas cis-5,8,11,14,17-Eicosapentaenoic acid (EPA), an ω-3 polyunsaturated fatty acid (PUFA) was purchased from Sigma‒Aldrich (purity >99%). Monoolein (MO, C18:1c9, powder, ≥99%), retinoic acid (RA), 2,6-di-tertbutyl-4-methylphenol (BHT), ginkgolide B, and quercetin were also purchased from Sigma‒Aldrich. The investigated peptide pituitary adenylate cyclase-activating polypeptide (PACAP) with molecular mass of 4535 Da, and purity of 99.48%), together with the rabies virus glycoprotein (RVG)-derived peptide conjugate (RVG-OL, MW 3659.30, charge +3 at pH 7, and purity 96.70%), and the RVG-Cy5.5 fluorescent conjugate were custom synthesized by QYAOBIO (China Peptides Co., Ltd., Rep. China). Phosphate buffer solution (NaH_2_PO_4_/Na_2_HPO_4_, 1×10^−2^ M; pH 7; p.a. grade; Merck) was prepared using Milli-Q quality (Millipore Corp., France)

#### Formulation of liquid crystalline lipid nanoparticles

Lyotropic liquid‒liquid crystalline nanoparticles were fabricated by hydrating a thin lipid film and subsequently dispersing it in an aqueous medium. Three distinct formulations were developed with mixed lipid compositions, which were additionally functionalized with PACAP and RVG-OL peptides: (i) LNP1, MO-EPA-Plu, (ii) LNP2, MO-EPA-Plu-GB-Quer + PACAP/RVG-OL, and (iii) LNP3, PL-EPA-Plu + PACAP (Supplementary Table 1). First, the lipids, surfactants, and active components were dissolved in chloroform. The solvent was removed by evaporation under a nitrogen flow at room temperature for 1 h to form a thin lipid layer, which was then lyophilized overnight to ensure complete solvent removal. The resulting dry films were rehydrated at room temperature in 1 × 10⁻² M phosphate buffer (pH 7.4) containing 2,6-di-tert-butyl-4-methylphenol (BHT) prepared using Milli-Q water. Before use, the phosphate buffer was degassed with nitrogen to remove dissolved oxygen and sterilized by filtration through a 0.2 μm membrane filter (Millipore, USA).

### In vitro experiments

#### Cell culture

SH-SY5Y human neuroblastoma cells were maintained in Dulbecco’s modified Eagle’s medium (DMEM; Sigma, France) supplemented with 10% (v/v) fetal bovine serum (FBS; Sigma) and 1% penicillin‒streptomycin. Cells were cultured at 37 °C in a humidified incubator with 5% CO₂. Cells were routinely passaged every 3 days, and experiments were performed using cells between passages 3 and 10. For neuronal differentiation, SH-SY5Y cells were seeded at approximately 5 × 10^6^ cells per flask and allowed to adhere for 24 h before they were treated with 10 µM retinoic acid (RA) for five days. Neuronal differentiation began when the cultures reached 70–80% confluence by replacing the culture medium with RA-supplemented medium. Cells were routinely monitored for morphology and tested periodically to confirm the absence of mycoplasma contamination.

#### Cytotoxicity and cell viability assays

SH-SY5Y human neuroblastoma cells were seeded into 96-well plates at a density of 1 × 10⁴ cells per well and incubated overnight at 37 °C under humidified conditions with 5% CO₂ to allow cell attachment. The following day, the medium was replaced with fresh complete medium supplemented with 10 µM retinoic acid (RA), and neuronal differentiation was carried out over five days with routine microscopic observation. Differentiated cells were then exposed to LCNPs at concentrations ranging from 1 to 400 µM for 24 h. RA-differentiated cells deprived of serum for 24 h (RA/FBS(–)) served as the control group. After treatment, cell-free supernatants (90 µL) were collected from each well and transferred to a new 96-well plate for the LDH assay. Subsequently, 10 µL of LDH Cytotoxicity Reagent from the CytoSelect™ LDH Cytotoxicity Assay Kit (CBA-241) was added to each sample and incubated for 30 min at 37 °C. Absorbance was then recorded at 450 nm using a microplate reader.

For MTT assay, the remaining adherent cells were incubated with 20 µL of freshly prepared MTT solution (5 mg/mL in PBS) for 1 h at 37 °C. The medium was discarded, and the resulting formazan crystals were solubilized in DMSO. The absorbance was measured at 570 nm. The cell viability is expressed as a percentage relative to that of the RA/FBS(–) control.

#### Cellular internalization of nanoparticulate assemblies

SH-SY5Y cells were plated in 6-well plates at a density of 3 × 10⁵ cells/well and differentiated for five days in the presence of 10 µM RA. Following differentiation, the medium was replaced with serum-free RA-containing medium supplemented with fluorescently labeled nanoassemblies, and the cells were incubated for predetermined time periods. After incubation, the cells were washed three times with ice-cold PBS and detached with an appropriate dissociation buffer. The cells were centrifuged at 200 × g for 5 min. The pelleted cells were reconstituted in 1 mL PBS for flow cytometry analysis (BD Accuri C6). Fluorescence was detected in the FL3 channel for free RVG-Cy5.5 fluorescent peptide and the LNP-RVG-Cy5.5 conjugate.

#### Quantification of phosphorylated AKT and ERK protein levels using ELISA

Levels of phosphorylated ERK1/2 and AKT were evaluated in SH-SY5Y cells following nanoparticle exposure using intracellular ELISA-based detection kits (Phospho-ERK1/2 DuoSet™ IC ELISA, Cat. #DYC1018B-5, and Phospho-AKT Pan Specific DuoSet® IC ELISA, Cat. #DYC887B-5; Bio-Techne/R&D Systems, UK). Cellular proteins were isolated using the extraction buffer included in the DuoSet® IC ancillary reagent kit (Cat. #DY008; Bio-Techne/R&D Systems, UK). 96-well plates precoated with monoclonal antibodies against p-AKT or p-ERK1/2 were incubated with protein standards or cell lysates for 2 h. Bound proteins were detected with enzyme-conjugated monoclonal antibodies, followed by a 1 h incubation at room temperature. After three washes, the substrate solution was added, and color development was stopped prior to measurement at 450 nm using a microplate reader. The signal intensity was proportional to the concentration of phospho- AKT and ERK.

#### Quantification of human BDNF using ELISA immunoassays

Human free BDNF levels were measured in SH-SY5Y cells following 24 h of exposure to nanoparticle treatment. Quantification was performed using a Human Free BDNF ELISA kit (Cat. #DBD00; R&D Systems; Bio-Techne, UK). Briefly, 96-well plates precoated with a monoclonal antibody specific for BDNF were loaded with 50 μL of standards, controls, or samples along with 100 μL of assay diluent and incubated for 2 h at room temperature. The wells were then treated with 100 μL of enzyme-linked anti-BDNF monoclonal antibody, followed by incubation for 1 h. After three washes to remove unbound reagents, 200 μL of substrate solution was added, and the samples were incubated for 30 min in the dark. The reaction was stopped with 50 μL of stop solution, and the absorbance was recorded at 450 nm within 30 min using a microplate reader.

#### Quantification of p38 MAPK and STAT3 phosphorylation

Phosphorylated levels of p38 MAPKα and p-STAT3 were measured using commercial sandwich ELISA kits (p38 MAPKα, Abcam ab221013) and STAT3 (pY705, Abcam ab126459) following the manufacturer’s instructions. SH-SY5Y cell lysates were prepared using lysis buffers supplemented with protease and phosphatase inhibitors, and the protein concentration was determined by a BCA assay. For both assays, an equal volume of cell lysate (100 µL) was loaded into 96-well plates precoated with capture antibodies against total p38 MAPKα or STAT3 (pY705). After incubation for 2.5 hours at room temperature, the wells were washed and then incubated with a phospho-specific antibody (p-p38 MAPKα Thr180/Tyr182 or p-STAT3 Tyr705). HRP-conjugated secondary antibodies were then applied for 1 hour, followed by the addition of TMB substrate. Colorimetric detection was performed at 450 nm using a microplate reader.

#### Mitochondrial superoxide

Mitochondrial superoxide production was successfully detected using mitochondrial superoxide dye (STEMCELL Technologies, Cat. #100-0991). The dye selectively localized to the mitochondria and produced a strong red fluorescence signal following oxidation by superoxide. Cells were seeded at a density of 3 × 10^5^ cells/well and treated with LNPs for 24 h. Subsequently, the cells were stained with mitochondrial superoxide dye, and fluorescence detection was carried out using fluorescence microscopy (FLUOstar OPTIMA microplate reader, Ex/Em = 510/580 nm), and the signal intensity correlated with the treatment conditions.

#### Lipid peroxidation (MDA)

Lipid peroxidation was quantified using a Lipid Peroxidation (MDA) Assay Kit (Sigma‒Aldrich, Cat. No. MAK085) according to the manufacturer’s protocol. For sample preparation, cells (5 × 10⁶) were homogenized on ice in 300 μL of MDA lysis buffer. supplemented with 3 μL of 100× butylated hydroxytoluene (BHT) to prevent artificial lipid oxidation. The homogenates were subsequently centrifuged at 13,000 × g for 10 minutes at 4 °C to remove insoluble debris, after which the supernatants were collected for analysis. For the assays, 200 μL of each sample or MDA standard was combined with 600 μL of freshly prepared TBA solution, vortexed, and incubated at 95 °C for 60 minutes to form the MDA-TBA adduct. The reaction mixtures were cooled on ice and centrifuged to remove particulates. The absorbance was measured at 540 nm using a microplate reader.

#### Animal model and treatment

Conditional mitochondrial transcription factor A (*Tfam*) knockout mice (10 weeks old; Strain No. T053276, GemPharmatech Co., Ltd.) were generated by crossing B6/JGpt-Tfam^em1Cflox/Gpt mice with B6/JGpt-Slc6a3^em1Cin(ires-iCre)/Gpt transgenic mice, resulting in the generation of a dopaminergic neuron–specific Tfam deletion (B6/JGpt-Tfam^em1CfloxSlc6a3^em1Cin(ires-iCre)/Gpt), which served as the Parkinson’s disease (PD) model, *i.e*., the MP model. Male C57BL/6JGpt mice (10 weeks old; Strain NO. N000013; GemPharmatech Co., Ltd.) were used as wild-type (WT) controls. Thirty-two adult male B6-Tfam-flox/Slc6a3-Cre and 8 WT mice were initially employed for this study. The transgenic and WT mice were randomly assigned to five experimental groups (*n* = 8 per group): (i) WT + PBS, (ii) MP model + PBS, (iii) MP model + LNP1, (iv) MP model + LNP2, and (v) MP model + LNP3. For treatment, mice in the LNP groups received intranasal administration of the respective LCNP formulation, with bilateral drops (20 μL for each nostril)administered through micropipette delivery once every week for three weeks. Intranasal administration was performed under light anesthesia. The mice were placed in a supine position with the head slightly elevated to facilitate nasal delivery. The total dose was administered as multiple small aliquots (5 µL per drop), allowing short intervals between drops to permit absorption and minimize swallowing or aspiration. The control groups received equivalent volumes of PBS. The delivered doses of each component were calculated from the formulation concentration (mg/mL) and dosing volume. The estimated mg/kg values were normalized to the average mouse body weight (~25 g) at treatment initiation and are summarized in Supplementary Table 2.

Only male mice were included in this study to reduce the variability associated with hormonal fluctuations. However, given the known sex differences in neuroinflammatory responses, future studies will be needed to evaluate LCNP formulation in both sexes to assess potential sex-dependent effects. Kaplan‒Meier survival curves were generated to evaluate survival. Each experimental group initially contained eight mice. Natural death (including animals found dead during monitoring) was considered an event. The animals were monitored daily throughout the study period, and their body weights were recorded every 3 days. Mice that remained alive at the end of the observation period were euthanized for tissue collection and treated as censored observations in the Kaplan‒Meier analysis. The mice were randomly allocated to treatment groups after baseline body weight assessment to ensure balanced group distribution. Behavioral testing and histological analyses were conducted under blinded conditions. Animals that died unexpectedly during the study were excluded from the endpoint analyses but were included in the survival evaluation where appropriate. All animal procedures were conducted in accordance with institutional guidelines and approved by the Institutional Animal Care and Use Committee (IACUC) of the Wenzhou Institute, University of Chinese Academy of Sciences (Approval No. GJS0420231201118).

#### Rotarod test

Motor coordination and balance were assessed using a rotarod apparatus over two consecutive days. On day 1 (training), the mice underwent three adaptation sessions consisting of 90 s on a stationary rod followed by 90 s of walking at 5 rpm/min. On day 2 (testing), the mice were tested on an accelerating rotarod (5 rpm/min to 40 rpm/min over 300 s). Each trial ended when all the mice either fell or reached the cutoff time, with three trials performed per mouse, and the mean latency to fall was recorded.

#### Buried food test

Olfactory function was evaluated using the buried food test. The mice were food deprived for 24 h prior to testing, with free access to water. Testing was conducted in a box (12 × 16 × 36 cm) containing 6 cm of clean corn cob bedding. After a 10 min habituation period, 2 g of food was buried in one corner of the box, while the mouse was placed in the opposite corner facing away from the food. The latency to locate and contact the food was recorded, with a maximum trial length of 600 s.

#### Immunofluorescence staining (ATP5A1 and α-Syn)

Formalin-fixed, paraffin-embedded tissue sections (4–6 μm) were deparaffinized in xylene (3 × 15 min), rehydrated in absolute ethanol (3 × 5 min) and distilled water, subjected to antigen retrieval in citrate buffer (pH 6.0) in a microwave (8 min, 8 min, 7 min), cooled, and then washed in PBS (pH 7.4). The slides were placed in 3% hydrogen peroxide solution and incubated at room temperature in the dark for 25 minutes to block endogenous peroxidase activity. Additionally, nonspecific binding was blocked with 3% BSA (Solarbio, A8020) or 10% rabbit serum for 30 min. Primary antibodies against α-synuclein and ATP synthase subunit α (ATP5A1, ZD396969, 1:400) were applied overnight at 4 °C in a humidified chamber, followed by incubation with an HRP-conjugated goat anti-rabbit secondary antibody (Abcam, ab205718, 1:5000). Signal amplification and visualization were performed using the 488-TSA Multiplex Staining Kit (Invitrogen, B40953). The nuclei were counterstained with DAPI (Wack, WK00020), and the slides were mounted with anti-fade mounting medium (Wack, WK11003). Images were acquired with a fluorescence scanner, where DAPI staining appeared blue and ATP5A1-positive signals appeared green.

#### Immunohistochemistry

IHC on paraffin sections was performed as described for IF (deparaffinization, antigen retrieval, PBS washes, peroxidase quenching, and blocking), followed by incubation with primary antibodies against IBA1 and TH (tyrosine hydroxylase) overnight at 4 °C in a humidified chamber. After the cells were washed with PBS (3 × 5 min), they were incubated with HRP-conjugated secondary antibodies specific to the primary antibody species for 50 min at room temperature. Freshly prepared DAB chromogenic solution was added, and color development was monitored under a microscope. The reaction was stopped by rinsing with water. Nuclei were counterstained with Harris hematoxylin for 3 min, washed, and differentiated briefly in 1% hydrochloric acid alcohol. The slides were dehydrated in ethanol (75% and 85% ethanol 5 min each, absolute ethanol 2 × 5 min), cleared in xylene, air-dried, and mounted with neutral balsam. Images were acquired using a light microscope, with nuclei appearing blue and positive IBA1 or TH expression appearing brown–yellow.

#### H&E staining of brain sections

Brain tissues (hippocampus, frontal cortex, substantia nigra, and cerebellum) from control and LNP-treated mice (*n* = 5 per group) were collected for hematoxylin and eosin (H&E) staining. The samples were dehydrated using a Donatello tissue processor (DIAPATH), embedded in paraffin with a JB-P5 embedding system, and sectioned at 5 μm thickness on an RM2016 rotary microtome. Sections were mounted onto HDAS001A glass slides, deparaffinized in xylene (Sinopharm, Cat. No. 10023418), and rehydrated through a graded ethanol series (75%, 85%, 95%). Staining was performed using a commercially available H&E staining kit (Solarbio, Cat. No. G1120). Tissue sections were subsequently visualized with a Nikon Eclipse E100 upright fluorescence microscope, and digital images were captured using a Nikon DS-U3 imaging system. The interpretation of the results was based on standard histological morphology, with the nuclei stained blue and the cytoplasm red, enabling the assessment of tissue structure and pathological alterations.

#### RNA-sequencing analysis: library preparation for transcriptome sequencing

RNA integrity was assessed using the RNA Nano 6000 Assay Kit on an Agilent 2100 Bioanalyzer (Agilent Technologies, USA). High-quality RNA was used for transcriptome library construction. Briefly, mRNA was purified from total RNA using poly-T oligo–attached magnetic beads, fragmented, and reverse transcribed into cDNA. After end repair, adaptor ligation, and size selection (~370–420 bp), the cDNA libraries were amplified by PCR and quality checked on an Agilent 2100 Bioanalyzer. Indexed libraries were clustered on a cBot Cluster Generation System (Illumina) and sequenced on the Illumina NovaSeq platform to generate 150 bp paired-end reads.^49,50^

#### RNA sequencing and bioinformatics analysis

The raw sequencing reads in FASTQ format were processed with *fastp* to remove adapters and low-quality sequences. Afterward, the clean reads were mapped to the mouse reference genome (GRCm39) using HISAT2 (v2.0.5), and transcript assembly was performed with StringTie (v1.3.3b). The gene-level counts were subsequently obtained using featureCounts (v1.5.0-p3) and normalized as fragments per kilobase of transcript per million mapped reads (FPKM). Differential expression analysis was conducted with DESeq2, with significance thresholds set at adjusted p ≤ 0.05 and log2-fold change ≥ 0.35. Functional enrichment pathway analysis (GO and KEGG) was performed with the clusterProfiler R package. GSEA was carried out with the Broad Institute GSEA tool using the Gene Ontology (GO) and Kyoto Encyclopedia of Genes and Genomes (KEGG) datasets.^51,52^ Additional analyses included single-nucleotide polymorphism (SNP) calling with GATK (v4.1.1.0), alternative splicing detection with rMATS (v4.1.0), protein–protein interaction (PPI) network construction using the STRING database, and weighted gene coexpression network analysis (WGCNA) to identify gene modules associated with phenotypes.^53–59^ For data visualization, Ggplot2 was used to generate volcano plots, donut plots, and boxplots of gene expression, whereas Pheatmap and Venndiagram were used to generate heatmaps and Venn diagrams (Supplementary Figs. 8-10).

#### Statistical analysis

Statistical analyses were carried out using GraphPad Prism version 9.5.1, with the results expressed as the mean ± SD. Independent Student’s *t* tests were used for two-group comparisons, while one-way and two-way ANOVA were employed for analyzing multiple groups. *p* values < 0.05 were considered to indicate statistical significance. Furthermore, the Sigmoidal 4PL model was utilized to evaluate the relationship between cellular responses and protein expression levels.

## Supplementary information

Sigtrans_Supplementary material

## Data Availability

All the data needed to evaluate the conclusions in this paper are presented in the paper and/or the Supplementary Materials. The RNA sequencing data generated in this study have been deposited in the NCBI database under BioProject accession number PRJNA1449156. Additional supporting data are available from the corresponding author upon reasonable request. The data that support the findings of this study are available from the corresponding author upon reasonable request. All data, materials, and methods are included in the article. Additional supporting data are available from the corresponding author upon reasonable request.

## References

[1] Lee, W. R. et al. Transcriptomic analysis of mitochondrial TFAM depletion changing cell morphology and proliferation. *Sci. Rep.* **7**, 17841 (2017). 29259235 10.1038/s41598-017-18064-9 PMC5736646

[2] Kaufman, B. A. et al. The Mitochondrial Transcription Factor TFAM Coordinates the Assembly of Multiple DNA Molecules into Nucleoid-like Structures. *Mol. Biol. Cell* **18**, 3225–3236 (2007). 17581862 10.1091/mbc.E07-05-0404 PMC1951767

[3] Picca, A. & Lezza, A. M. S. Regulation of mitochondrial biogenesis through TFAM–mitochondrial DNA interactions. *Mitochondrion* **25**, 67–75 (2015). 26437364 10.1016/j.mito.2015.10.001

[4] Schapira, A. H. Mitochondria in the etiology and pathogenesis of Parkinson’s disease. *Lancet Neurol* **7**, 97–109 (2008). 18093566 10.1016/S1474-4422(07)70327-7

[5] Ekstrand, M. I. et al. Progressive parkinsonism in mice with respiratory-chain-deficient dopamine neurons. *Proc. Natl. Acad. Sci.* **104**, 1325–1330 (2007). 17227870 10.1073/pnas.0605208103 PMC1783140

[6] Ekstrand, M. I. & Galter, D. The MitoPark Mouse – An animal model of Parkinson’s disease with impaired respiratory chain function in dopamine neurons. *Parkinsonism Relat. Disord.* **15**, S185–S188 (2009). 20082987 10.1016/S1353-8020(09)70811-9

[7] Galter, D. et al. MitoPark mice mirror the slow progression of key symptoms and L-DOPA response in Parkinson’s disease. *Genes Brain Behav* **9**, 173–181 (2010). 20002202 10.1111/j.1601-183X.2009.00542.x PMC4154513

[8] Li, X. et al. Cognitive Dysfunction Precedes the Onset of Motor Symptoms in the MitoPark Mouse Model of Parkinson’s Disease. *PLoS ONE* **8**, e71341 (2013). 23977020 10.1371/journal.pone.0071341 PMC3744561

[9] Beckstead, M. J. & Howell, R. D. Progressive parkinsonism due to mitochondrial impairment: Lessons from the MitoPark mouse model. *Exp. Neurol.* **341**, 113707 (2021). 33753138 10.1016/j.expneurol.2021.113707 PMC8169575

[10] Langley, M. et al. Mito-Apocynin Prevents Mitochondrial Dysfunction, Microglial Activation, Oxidative Damage, and Progressive Neurodegeneration in MitoPark Transgenic Mice. *Antioxid. Redox Signal.* **27**, 1048–1066 (2017). 28375739 10.1089/ars.2016.6905 PMC5651937

[11] Ay, M. et al. Mito-metformin protects against mitochondrial dysfunction and dopaminergic neuronal degeneration by activating upstream PKD1 signaling in cell culture and MitoPark animal models of Parkinson’s disease. *Front. Neurosci.* **18**, 1356703 (2024). 38449738 10.3389/fnins.2024.1356703 PMC10915001

[12] Maigler, F. et al. Selective CNS Targeting and Distribution with a Refined Region-Specific Intranasal Delivery Technique via the Olfactory Mucosa. *Pharmaceutics* **13**, 1904 (2021). 34834319 10.3390/pharmaceutics13111904 PMC8620656

[13] Akanchise, T. & Angelova, A. Potential of Nano-Antioxidants and Nanomedicine for Recovery from Neurological Disorders Linked to Long COVID Syndrome. *Antioxidants* **12**, 393 (2023). 36829952 10.3390/antiox12020393 PMC9952277

[14] Akanchise, T., Angelov, B. & Angelova, A. Nanomedicine-mediated recovery of antioxidant glutathione peroxidase activity after oxidative-stress cellular damage: Insights for neurological long COVID. *J. Med. Virol.* **96**, e29680 (2024). 38767144 10.1002/jmv.29680

[15] Fujino, T. et al. Efficacy and Blood Plasmalogen Changes by Oral Administration of Plasmalogen in Patients with Mild Alzheimer’s Disease and Mild Cognitive Impairment: A Multicenter, Randomized, Double-blind, Placebo-controlled Trial. *eBioMedicine* **17**, 199–205 (2017). 28259590 10.1016/j.ebiom.2017.02.012 PMC5360580

[16] Derosa, G., Maffioli, P., D’Angelo, A. & Di Pierro, F. A role for quercetin in coronavirus disease 2019 (COVID-19). *Phytother. Res.* **35**, 1230–1236 (2021). 33034398 10.1002/ptr.6887 PMC7675685

[17] Boots, A. W., Drent, M., de Boer, V. C. J., Bast, A. & Haenen, G. R. M. M. Quercetin reduces markers of oxidative stress and inflammation in sarcoidosis. *Clin. Nutr.* **30**, 506–512 (2011). 21324570 10.1016/j.clnu.2011.01.010

[18] Barbalho, S. M. et al. *Ginkgo biloba* in the Aging Process: A Narrative Review. *Antioxidants* **11**, 525 (2022). 35326176 10.3390/antiox11030525 PMC8944638

[19] Gachowska, M., Szlasa, W., Saczko, J. & Kulbacka, J. Neuroregulatory role of ginkgolides. *Mol. Biol. Rep.* **48**, 5689–5697 (2021). 34245409 10.1007/s11033-021-06535-2 PMC8338821

[20] Xiao, Q. et al. Ginkgolide B protects hippocampal neurons from apoptosis induced by beta-amyloid 25–35 partly via upregulation of brain-derived neurotrophic factor. *Eur. J. Pharmacol.* **647**, 48–54 (2010). 20709055 10.1016/j.ejphar.2010.08.002

[21] Akanchise, T. & Angelova, A. Ginkgo Biloba and Long COVID: In Vivo and In Vitro Models for the Evaluation of Nanotherapeutic Efficacy. *Pharmaceutics* **15**, 1562 (2023). 37242804 10.3390/pharmaceutics15051562 PMC10224264

[22] Ashok, A. et al. Antioxidant Therapy in Oxidative Stress-Induced Neurodegenerative Diseases: Role of Nanoparticle-Based Drug Delivery Systems in Clinical Translation. *Antioxidants* **11**, 408 (2022). 35204290 10.3390/antiox11020408 PMC8869281

[23] Che, H. et al. A comparative study of EPA-enriched ethanolamine plasmalogen and EPA-enriched phosphatidylethanolamine on Aβ_42_ induced cognitive deficiency in a rat model of Alzheimer’s disease. *Food Funct* **9**, 3008–3017 (2018). 29774334 10.1039/c8fo00643a

[24] Yang, T.-X. et al. EPA-enriched plasmalogen attenuates the cytotoxic effects of LPS-stimulated microglia on the SH-SY5Y neuronal cell line. *Brain Res. Bull.* **186**, 143–152 (2022). 35728742 10.1016/j.brainresbull.2022.06.002

[25] Shen, H.-H. et al. The influence of dipalmitoyl phosphatidylserine on phase behavior of and cellular response to lyotropic liquid crystalline dispersions. *Biomaterials* **31**, 9473–9481 (2010). 20880581 10.1016/j.biomaterials.2010.08.030

[26] Clementino, A. R. et al. Structure and Fate of Nanoparticles Designed for the Nasal Delivery of Poorly Soluble Drugs. *Mol. Pharm.* **18**, 3132–3146 (2021). 34259534 10.1021/acs.molpharmaceut.1c00366 PMC8335725

[27] Wu, Y. et al. Sustained CREB phosphorylation by lipid-peptide liquid crystalline nanoassemblies. *Commun. Chem.* **6**, 241 (2023). 37932487 10.1038/s42004-023-01043-9 PMC10628290

[28] Boyd, B. J., Whittaker, D. V., Khoo, S.-M. & Davey, G. Lyotropic liquid crystalline phases formed from glycerate surfactants as sustained release drug delivery systems. *Int. J. Pharm.* **309**, 218–226 (2006). 16413980 10.1016/j.ijpharm.2005.11.033

[29] Wang, Q. et al. Efficient Sustained-Release Nanoparticle Delivery System Protects Nigral Neurons in a Toxin Model of Parkinson’s Disease. *Pharmaceutics* **14**, 1731 (2022). 36015354 10.3390/pharmaceutics14081731 PMC9415969

[30] Kongkatigumjorn, N. et al. Controlling Endosomal Escape Using pH-Responsive Nanoparticles with Tunable Disassembly. *ACS Appl. Nano Mater.* **1**, 3164–3173 (2018).

[31] Akanchise, T. et al. Rapid structural transformation of ionizable lipid nanoparticles involving Omega-3 polyunsaturated fatty acids enhances antioxidant defense and mitochondrial proteins activity in pH-responsive drug delivery. *J. Colloid Interface Sci.* **704**, 139420 (2026). 41223481 10.1016/j.jcis.2025.139420

[32] Fakhri, S. et al. Nanoparticles in Combating Neuronal Dysregulated Signaling Pathways: Recent Approaches to the Nanoformulations of Phytochemicals and Synthetic Drugs Against Neurodegenerative Diseases. *Int. J. Nanomedicine* **17**, 299–331 (2022). 35095273 10.2147/IJN.S347187 PMC8791303

[33] Kundu, P., Das, M., Tripathy, K. & Sahoo, S. K. Delivery of Dual Drug Loaded Lipid Based Nanoparticles across the Blood–Brain Barrier Impart Enhanced Neuroprotection in a Rotenone Induced Mouse Model of Parkinson’s Disease. *ACS Chem. Neurosci.* **7**, 1658–1670 (2016). 27642670 10.1021/acschemneuro.6b00207

[34] Badoiu, S. C. et al. PI3K/AKT/mTOR Dysregulation and Reprogramming Metabolic Pathways in Renal Cancer: Crosstalk with the VHL/HIF Axis. *Int. J. Mol. Sci.* **24**, 8391 (2023). 37176098 10.3390/ijms24098391 PMC10179314

[35] Gao, Y. et al. RVG-Peptide-Linked Trimethylated Chitosan for Delivery of siRNA to the Brain. *Biomacromolecules* **15**, 1010–1018 (2014). 24547943 10.1021/bm401906p

[36] Smit, R. D. et al. STAT3 protects dopaminergic neurons against degeneration in animal model of Parkinson’s disease. *Brain Res* **1824**, 148691 (2024). 38030102 10.1016/j.brainres.2023.148691 PMC10842767

[37] Barry, S. P. et al. STAT3 deletion sensitizes cells to oxidative stress. *Biochem. Biophys. Res. Commun.* **385**, 324–329 (2009). 19450559 10.1016/j.bbrc.2009.05.051 PMC2706948

[38] Cheng, X., Peuckert, C. & Wölfl, S. Essential role of mitochondrial Stat3 in p38MAPK mediated apoptosis under oxidative stress. *Sci. Rep.* **7**, 15388 (2017). 29133922 10.1038/s41598-017-15342-4 PMC5684365

[39] Hanson, L. R. & Frey, W. H. Intranasal delivery bypasses the blood‒brain barrier to target therapeutic agents to the central nervous system and treat neurodegenerative disease. *BMC Neurosci* **9**, S5 (2008). 19091002 10.1186/1471-2202-9-S3-S5 PMC2604883

[40] Van Vliet, E. F., Knol, M. J., Schiffelers, R. M., Caiazzo, M. & Fens, M. H. A. M. Levodopa-loaded nanoparticles for the treatment of Parkinson’s disease. *J. Controlled Release* **360**, 212–224 (2023). 10.1016/j.jconrel.2023.06.026 37343725

[41] Di Francesco, V., Chua, A. J., Bleier, B. S. & Amiji, M. M. Effective Nose-to-Brain Delivery of Blood-Brain Barrier Impermeant Anti-IL-1β Antibody via the Minimally Invasive Nasal Depot (MIND) Technique. *ACS Appl. Mater. Interfaces* **16**, 69103–69113 (2024). 39655527 10.1021/acsami.4c18679 PMC11660045

[42] Rey, N. L. et al. Spread of aggregates after olfactory bulb injection of α-synuclein fibrils is associated with early neuronal loss and is reduced long term. *Acta Neuropathol. (Berl.)* **135**, 65–83 (2018). 29209768 10.1007/s00401-017-1792-9 PMC5756266

[43] Asimakidou, E., Tan, J. K. S., Zeng, J. & Lo, C. H. Blood‒Brain Barrier-Targeting Nanoparticles: Biomaterial Properties and Biomedical Applications in Translational Neuroscience. *Pharm. Basel Switz.* **17**, 612 (2024). 10.3390/ph17050612 PMC11123901 38794182

[44] Dighe, S., Jog, S., Momin, M., Sawarkar, S. & Omri, A. Intranasal Drug Delivery by Nanotechnology: Advances in and Challenges for Alzheimer’s Disease Management. *Pharmaceutics* **16**, 58 (2023). 38258068 10.3390/pharmaceutics16010058 PMC10820353

[45] Hersh, A. M., Alomari, S. & Tyler, B. M. Crossing the Blood‒Brain Barrier: Advances in Nanoparticle Technology for Drug Delivery in Neuro-Oncology. *Int. J. Mol. Sci.* **23**, 4153 (2022). 35456971 10.3390/ijms23084153 PMC9032478

[46] Liu, S. et al. Advances in brain-targeted delivery strategies and natural product-mediated enhancement of blood–brain barrier permeability. *J. Nanobiotechnology* **23**, 382 (2025). 40420216 10.1186/s12951-025-03415-w PMC12107825

[47] Meng, Q. et al. Influence of nanoparticle size on blood–brain barrier penetration and the accumulation of anti-seizure medicines in the brain. *J. Mater. Chem. B* **10**, 271–281 (2022). 34897348 10.1039/d1tb02015c

[48] O’Connor, D. M. & Boulis, N. M. Gene therapy for neurodegenerative diseases. *Trends Mol. Med.* **21**, 504–512 (2015). 26122838 10.1016/j.molmed.2015.06.001

[49] Mortazavi, A., Williams, B. A., McCue, K., Schaeffer, L. & Wold, B. Mapping and quantifying mammalian transcriptomes by RNA-Seq. *Nat. Methods* **5**, 621–628 (2008). 18516045 10.1038/nmeth.1226 PMC13303166

[50] Wang, Z., Gerstein, M. & Snyder, M. RNA-Seq: a revolutionary tool for transcriptomics. *Nat. Rev. Genet.* **10**, 57–63 (2009). 19015660 10.1038/nrg2484 PMC2949280

[51] Yu, G., Wang, L.-G., Yan, G.-R. & He, Q.-Y. DOSE: an R/Bioconductor package for disease ontology semantic and enrichment analysis. *Bioinformatics* **31**, 608–609 (2015). 25677125 10.1093/bioinformatics/btu684

[52] Yu, G., Wang, L.-G., Han, Y. & He, Q.-Y. clusterProfiler: an R Package for Comparing Biological Themes Among Gene Clusters. *OMICS J. Integr. Biol.* **16**, 284–287 (2012). 10.1089/omi.2011.0118 PMC3339379 22455463

[53] Pertea, M. et al. StringTie enables improved reconstruction of a transcriptome from RNA-seq reads. *Nat. Biotechnol.* **33**, 290–295 (2015). 25690850 10.1038/nbt.3122 PMC4643835

[54] Love, M. I., Huber, W. & Anders, S. Moderated estimation of fold change and dispersion for RNA-seq data with DESeq2. *Genome Biol*. **15**, (2014). 10.1186/s13059-014-0550-8 PMC4302049 25516281

[55] Liao, Y., Smyth, G. K. & Shi, W. featureCounts: an efficient general purpose program for assigning sequence reads to genomic features. *Bioinformatics* **30**, 923–930 (2014). 24227677 10.1093/bioinformatics/btt656

[56] Young, M. D., Wakefield, M. J., Smyth, G. K. & Oshlack, A. Gene ontology analysis for RNA-seq: accounting for selection bias. *Genome Biol*. **11**, (2010). 10.1186/gb-2010-11-2-r14 PMC2872874 20132535

[57] He, Z. et al. Comparative transcriptome and gene coexpression network analysis reveal genes and signaling pathways adaptively responsive to varied adverse stresses in the insect fungal pathogen, Beauveria bassiana. *J. Invertebr. Pathol.* **151**, 169–181 (2018). 29258843 10.1016/j.jip.2017.12.002

[58] McKenna, A. et al. The Genome Analysis Toolkit: A MapReduce framework for analyzing next-generation DNA sequencing data. *Genome Res* **20**, 1297–1303 (2010). 20644199 10.1101/gr.107524.110 PMC2928508

[59] Shen, S. et al. rMATS: Robust and flexible detection of differential alternative splicing from replicate RNA-Seq data. *Proc. Natl. Acad. Sci*. **111**, (2014). 10.1073/pnas.1419161111 PMC4280593 25480548

[60] Shimshek, D. R. et al. Codon-improved Cre recombinase (iCre) expression in the mouse. *Genesis* **32**, 19–26 (2002). 11835670 10.1002/gene.10023

