# Supplementary material for: Peptide-targeted cubosome and hexosome nanoassemblies mitigate mitochondrial dysfunction in a MitoPark model
Source: Signal Transduct Target Ther. 2026 Aug 4;11:310. doi: 10.1038/s41392-026-02897-w (PMC13434212; doi:10.1038/s41392-026-02897-w)
Supplement: Supplementary file 1 [file 41392_2026_2897_MOESM1_ESM.docx]

*Supplementary Materials for*

**Peptide-targeted cubosome and hexosome nanoassemblies mitigate mitochondrial dysfunction in a MitoPark model**

*Thelma Akanchise^1^, Fucen Luo^1^, Borislav Angelov^2^,* [*Yuru Deng*](https://loop.frontiersin.org/people/902111)*^3,4,*^, Takehiko Fujino*^5^ *and Angelina Angelova^1,*^*

*^*^*Corresponding authors’ e-mail: angelina.angelova@universite-paris-saclay.fr (A.A.);

dengyr@wiucas.ac.cn (Y.D.)

**Materials and Methods**

**Figure S1. Biodistribution of Cy5.5-labeled lipid nanoparticles following intranasal administration.** (a) Representative *in vivo* fluorescence images of mice at 0, 2, 4, 6, and 8 h after intranasal administration of Cy5.5-labeled LNPs (LNP+Cy5.5) or free Cy5.5 fluorescent dye. (b) Fluorescence images of major organs (brain, heart, liver, spleen, lung, and kidney) at 8 h after intranasal administration of the Cy5.5-labeled lipid nanoparticles or the free Cy5.5 dye to mice. The images were collected at the indicated time point following intranasal administration of LNP+Cy5.5 or free Cy5.5, demonstrating differential tissue distribution. (c) Quantification of total fluorescent intensity in the brain region over time following intranasal administration. The LNP-encapsulated Cy5.5 exhibited prolonged signal retention compared with the free Cy5.5 marker. Data are presented as mean ± SD. n=2, *p < 0.05.

**Figure. S2. *In vivo* behavioural (rotarod and buried food tests), establishing the functional outcome and therapeutic benefits of the PUFA-enriched lipid nanoassemblies.** The results show **behavioural assessment** of motor and olfactory function in B6-Tfam-flox/Slc6a3-Cre-PD mouse model (MitoPark) after intranasal LNP treatment. (a,b) Rotarod test performance before (*n*=8) and after 3 weeks of treatment with LNP1, LNP2, and LNP3 (*n*=5). (c,d) Buried food test assessing olfactory function performed before (*n*=8) and after 3 weeks of treatment with LNP1, LNP2, and LNP3 (*n*=5). Data are presented as mean ± SD (ns > 0.05,****p < 0.0001). Statistical differences were calculated using Dunnett's multiple comparisons test for one‐way ANOVA.

**Figure. S3. α-Synuclein immunofluorescence in MitoPark mice and LNP-treated groups.** (a) Representative coronal brain sections showing α-synuclein (α-syn, green) immunofluorescence with DAPI nuclear counterstaining (blue) in control, MitoPark (MP) model, and LNP-treated groups (LNP1–3). Merged images are shown in the bottom row. (b) Quantification of relative α-synuclein-positive signal normalized to control levels. No statistically significant differences were detected between groups. Data are presented as mean ± SD with individual data points representing biological replicates; ns, not significant.

**Figure. S4. IBA1 immunohistochemistry in cerebellum and frontal cortex.** (a) Representative IBA1-stained coronal brain sections from control, MitoPark model, and LNP-treated groups showing microglial distribution in the cerebellum and frontal cortex. (b,c) Quantification of relative IBA1-positive signal in the cerebellum (b) and frontal cortex (c). Data is presented as relative values normalized to the control group. No statistically significant differences were observed between groups following LNP treatment. Individual data points represent biological replicates. Results are reported as mean ± SD (*n* = 5, *ns* > 0.05, *p < 0.5). Statistical differences were calculated using Dunnett's multiple comparisons test for one‐way ANOVA.

**Figure. S5. Category gene network of enriched KEGG pathways in MP versus LNP groups.** Using the same DEG set (gene symbol, padj ≤ 0.25), significant KEGG pathways (FDR ≤ 0.04) were visualized with cnetplot to illustrate gene membership within each pathway. Category node size corresponds to the number of genes, while gene node color represents log2 fold change (red = upregulated, green = downregulated). Edges indicate the annotation of a gene to the respective pathway.

**Figure. S6. KEGG pathway enrichment analysis of differentially expressed genes (DEGs) in MP versus LNP-treated groups.** Dot plots show significantly enriched KEGG pathways among upregulated (a, c, e) and downregulated (b, d, f) genes in comparisons of MP versus LNP1 (a–b), MP versus LNP2 (c–d), and MP versus LNP3 (e–f). The x-axis represents the GeneRatio (proportion of DEGs involved in each pathway), while the y-axis lists the enriched pathways. The size of the dots corresponds to the number of genes enriched in the pathway, and the color scale indicates adjusted p-values (padj), with red denoting higher significance.

**Figure. S7. Gene Set Enrichment Analysis (GSEA) reveals distinct pathway regulation in MP and LNP-treated groups.** (a) In the MP group, enriched pathways included AMPK signaling, apoptosis, MAPK signaling, JAK-STAT signaling, NF-κB signaling, PI3K-Akt signaling, p53 signaling, and ECM–receptor interaction. (b) The LNP2 group shows enriched pathways associated with neurodegenerative diseases such as Alzheimer’s disease, Huntington’s disease, and multiple neurodegeneration-related pathways. (c) In the LNP3 group, enrichment was observed for metabolic and protein regulation pathways, including the citrate cycle (TCA), pyruvate metabolism, and protein processing in the endoplasmic reticulum. (d) In the LNP1 group, pathways related to drug metabolism (cytochrome P450) and peroxisome function were significantly enriched.

**Figure. S8. Heatmap analysis of differentially expressed genes between MP and LNP-treated groups.** Hierarchical clustering heatmaps show the expression profiles of DEGs in (a) MP vs. LNP1, (b) MP vs. LNP2, and (c) MP vs. LNP3 groups. Each column represents an individual sample, and each row represents a gene. The color scale indicates relative expression levels (red = upregulation; blue = downregulation). Clear clustering patterns demonstrate distinct transcriptional differences between MP and LNP-treated groups.

**Figure. S9. Pearson correlation analysis of transcriptomic profiles in MP and LNP groups.**
(a) Pearson correlation heatmap showing the similarity of global expression profiles between MP, LNP1, LNP2, and LNP3 groups. (b) Inter-sample Pearson correlation heatmap across all replicates within each group. Correlation strength is indicated by the color scale (R²), with values close to 1 representing highly similar transcriptomic patterns. Only genes meeting the DEG selection cutoff (padj ≤ 0.05, log2FC ≥ 0.35) were included in the analysis. G6, G7_1, G7_2, and G7_3 correspond to MP, LNP1, LNP2, and LNP3 samples, respectively.

**Figure. S10. An overview of gene expression overlap and distribution across MP and LNP groups.** (a) Venn diagrams showing the overlap of expressed genes between MP and each LNP treatment group (LNP1, LNP2, LNP3). Numbers represent the count of uniquely and commonly expressed genes in each comparison. (b) Boxplots of log2(FPKM + 1) values across all samples in MP (G6) and LNP-treated groups (G7_1 = LNP1, G7_2 = LNP2, G7_3 = LNP3). The distribution of expression values indicates consistent normalization and comparable transcriptome-wide expression across samples.

**Table S1. Amphiphilic compositions of lipid nanoparticle formulations used for *in vivo* studies.** Formulations (LNP1, LNP2, and LNP3) were prepared with varying amphiphilic components, including monoolein (MO), plasmalogen (PL), eicosapentaenoic acid (EPA), ginkgolide B, quercetin, and stabilizer Pluronic F127. Functionalization with the peptides PACAP (MW 4535 Da, purity 99.48%) and RVG-OL conjugate (MW 3659.30 Da, purity 96.70%) was employed in chosen cases to facilitate neuronal targeting and "homing", enhance the neuroprotective efficacy, and cellular uptake. The peptide concentration in the formulations corresponds to 1 mg/mL.

| **Sample code** | **Multi-target formulations prepared for *in vivo* studies by  self-assembly of lipid nanoparticles with the peptides PACAP and RVG-OL** |
| --- | --- |
| **LNP1** | MO - EPA - PluronF127 |
| **LNP2** | MO-EPA-GinkgolideB-Quercetin-PluronF127 + **PACAP** + **RVG-OL** |
| **LNP3** | PL-EPA-PluronF127 + **PACAP** |

**Table S2. Estimated intranasal dose per mouse of the LNP formulation components. The table summarizes the lipid and active-ingredient contents in each formulation and the corresponding estimated amount delivered per mouse following intranasal administration (20 µL per nostril; 40 µL total). Approximate** mg/kg doses were calculated based on the **measured body weight of mice at the onset of treatment (the mean body weight was 25 g)**. The peptide concentration in the formulations corresponds to 1 mg/mL. The antioxidants are incorporated at GinkgolideB (5%wt) and Quercetin (3%wt), and the dispersing agent at Pluronic F127(10%wt) with regard to the lipid component.

| **Formulation** | **Component** | **Amount per mouse (40 µL)** | **Dose (mg/kg)** |
| --- | --- | --- | --- |
| **LNP1** | Monoolein | 0.856 mg | 34.2 |
|  | EPA | 0.172 mg | 6.9 |
| **LNP2** | Monoolein | 0.856 mg | 34.2 |
|  | EPA | 0.172 mg | 6.9 |
|  | Ginkgolide B | 0.060 mg | 2.4 |
|  | Quercetin | 0.0256 mg | 1.0 |
|  | **PACAP** | 0.040 mg | 1.6 |
|  | **RVG-OL** | 0.040 mg | 1.6 |
| **LNP3** | Plasmalogen | 0.800 mg | 32 |
|  | EPA | 0.080 mg | 3.2 |
|  | **PACAP** | 0.040 mg | 1.6 |
